# Functional Characterization of *Pseudomonas* Contact Dependent Growth Inhibition (CDI) Systems

**DOI:** 10.1371/journal.pone.0147435

**Published:** 2016-01-25

**Authors:** Chryslène Mercy, Bérengère Ize, Suzana P. Salcedo, Sophie de Bentzmann, Sarah Bigot

**Affiliations:** 1 Laboratoire d’Ingénierie des Systèmes Macromoléculaires, CNRS UMR7255, Université Aix Marseille, Marseille, France; 2 Bases Moléculaires et Structurales des Systèmes Infectieux, CNRS UMR 5086, Université Lyon 1, Institut de Biologie et Chimie des Protéines, Lyon, France; University of North Dakota, UNITED STATES

## Abstract

Contact-dependent inhibition (CDI) toxins, delivered into the cytoplasm of target bacterial cells, confer to host strain a significant competitive advantage. Upon cell contact, the toxic C-terminal region of surface-exposed CdiA protein (CdiA-CT) inhibits the growth of CDI^-^ bacteria. CDI^+^ cells express a specific immunity protein, CdiI, which protects from autoinhibition by blocking the activity of cognate CdiA-CT. CdiA-CT are separated from the rest of the protein by conserved peptide motifs falling into two distinct classes, the “*E*. *coli*”- and “*Burkholderia*-type”. CDI systems have been described in numerous species except in Pseudomonadaceae. In this study, we identified functional toxin/immunity genes linked to CDI systems in the *Pseudomonas* genus, which extend beyond the conventional CDI classes by the variability of the peptide motif that delimits the polymorphic CdiA-CT domain. Using *P*. *aeruginosa* PAO1 as a model, we identified the translational repressor RsmA as a negative regulator of CDI systems. Our data further suggest that under conditions of expression, *P*. *aeruginosa* CDI systems are implicated in adhesion and biofilm formation and provide an advantage in competition assays. All together our data imply that CDI systems could play an important role in niche adaptation of Pseudomonadaceae.

## Introduction

Bacteria share habitats and have therefore developed cooperative and antagonistic behaviors that facilitate their struggle for living space and nutrient availability. To compete with each other, bacteria have evolved sophisticated weapons such as the polymorphic toxin systems to impede the proliferation of other microorganisms [1,2]. Polymorphic toxin systems are characterized by the presence of an immunity gene immediately downstream of the toxin gene whose product protects bacteria against suicide and fratricide. One of the major pathways for delivery of polymorphic toxins is the Contact-Dependent growth Inhibition (CDI) system, which is implicated in inter-bacterial competition and allows growth inhibition of neighboring competitors via direct cell-cell contact [3].

CDI systems are composed of the CdiA toxin secreted by CdiB, an outer membrane β-barrel transporter, and a CdiI immunity protein. CdiA and CdiB are part of the Two-Partner Secretion (TPS) system family. CdiA are very large filamentous proteins predicted to extend far from the cell surface [4]. CdiA homologs share large N-terminal sequences of homology that exhibit haemagglutinin repeats while their C-terminal domains, called CdiA-CT, are highly variable [5]. CdiA-CT carry toxin activity and are delivered to the cytoplasm of the target cell where they degrade DNA or specific tRNAs leading to growth inhibition or death. When present in target cells, cognate CdiI immunity proteins protect bacteria by specifically interacting with CdiA-CT and subsequently blocking their nuclease activity [5–7]. CdiI proteins are also highly variable and share no sequence homology, thus they confer immunity to their cognate toxin but not to toxins deployed by other CDI systems. The peptide motifs delimiting the CdiA-CT from the rest of the protein fall into two distinct classes, the “*E*. *coli*-type” and the “*Burkholderia*-type” restricted to *Burkholderia* species and a few closely related species of *Ralstonia* and *Cupriavidus* [5,8,9]. The genetic organization of the “*E*. *coli*”-type CDI locus is *cdiBAI* and the CdiA-CT regions diverge abruptly after a common VENN motif (PF04829) [5]. In *Burkholderia*, the CDI system operon is organized as *bcpAIOB* (*B**urkholderia* CDI proteins A, I, O and B), with the *bcpO* gene encoding a predicted lipoprotein required for BcpA function. The sequence divergence of the BcpA-CT toxic domain is observed after a conserved Nx(Q/E)LYN motif instead of the VENN motif [8,9].

In addition to mediating inter-bacterial competition, CDI systems are thought to play a role in biofilms establishment. Indeed, the CDI systems of *Burkholderia thailandensis* E264 and *E*. *coli* EC93 are implicated in auto-aggregation, adherence to abiotic surfaces and are required for biofilm formation [8,10,11]. The contribution to biofilm development might facilitate cooperative bacterial behaviors and eventually lead to an exclusion of non-self competitors from the community [12].

Mechanisms and functions of CDI systems have been well studied in Enterobacteria and *Burkholderia* species, but remain poorly characterized in Pseudomonadaceae. The Pseudomonadaceae includes a heterogeneous set of microorganisms that are able to colonize diverse niches, ranging from terrestrial and aquatic environments to tissues of eukaryotic hosts. To date over 100 different strains have been described with *Pseudomonas aeruginosa* being the best characterized so far. *P*. *aeruginosa* is the primary agent of opportunistic infection in humans, especially in cystic fibrosis (CF) patients, causing both acute and chronic infections. Ghequire and de Mot recently proposed putative CDI gene loci in Pseudomonadaceae through *in silico* analysis [13]. In this work, we show that predicted CDI loci are indeed widely distributed in *Pseudomonas* strains and that the pre-toxin motifs fall into at least five distinct classes including the “*E*. *coli*-type”. We functionally characterize two CDI systems present in *P*. *aeruginosa* PAO1 and show their negative regulation by the RNA-binding protein RsmA. We demonstrate that besides contributing to biofilm formation, these systems also mediate inter-bacterial competition between *Pseudomonas*. Furthermore we were able to demonstrate that the CdiA-CT domains carry toxic activities and are specifically blocked by their cognate CdiI immunity proteins. Finally, we extend this study showing that *P*. *aeruginosa* PA7, PA14 and *P*. *syringae* pv. tomato DC3000 strains also produce functional CdiA-CT toxin / CdiI immunity pairs.

## Results

### Identification of two potential CDI systems in *P*. *aeruginosa* PAO1 strain

*In silico* analysis of *P*. *aeruginosa* PAO1 genome revealed that the PA2463-PA2462 and PA0040-PA0041 loci contain characteristic features of a CDI locus. First, PA2463 and PA0040 are predicted to encode TpsB homologues whereas PA2462 and PA0041 contain the conserved TPS domain of TpsA proteins and encode putative large proteins of 570 and 360 kDa respectively. Moreover, PA2462 C-terminal (CT) domain shares 60% identity with MafB_MGI-NEM8013_, the bacterial EndoU ribonuclease recently identified in *Neisseria meningitidis* NEM8013 (S1 Fig and [14]) while 3D modelization of PA0041 CT domain revealed a predicted structure similar to the *Burkholderia pseudomallei* 1026b CdiA-CT ribonuclease domain [6]. We further noticed the presence of small non-annotated ORFs immediately downstream of PA2462 and PA0041, which potentially encode immunity proteins (S2 Fig). Additionally, both loci are flanked by transposable elements (data not shown) and the GC content decreases abruptly below 50% in the region encoding the CT domains and potential immunity proteins (S2 Fig) indicating that these regions might have been acquired by horizontal gene transfer, a characteristic of CDI systems. Interestingly, these clusters share high sequence identity (>95%) in the center and at the 5’ border (S2 Fig). By contrast, CT domain and immunity-encoding sequences share no homology in accordance with the high variability of CdiA-CT/CdiI modules. Based on the similarities with identified CDI systems, we renamed PA2463/PA0040, PA2462/PA0041, and predicted immunity proteins as CdiB_PA2463_/CdiB_PA0040_, CdiA_PA2462_/CdiA_PA0041_ and CdiI_PA2462_/CdiI_PA0041_ respectively.

### Expression and regulation of the *cdiA*_*PA2462*_ and *cdiA*_*PA0041*_ genes

**Fig 1:**
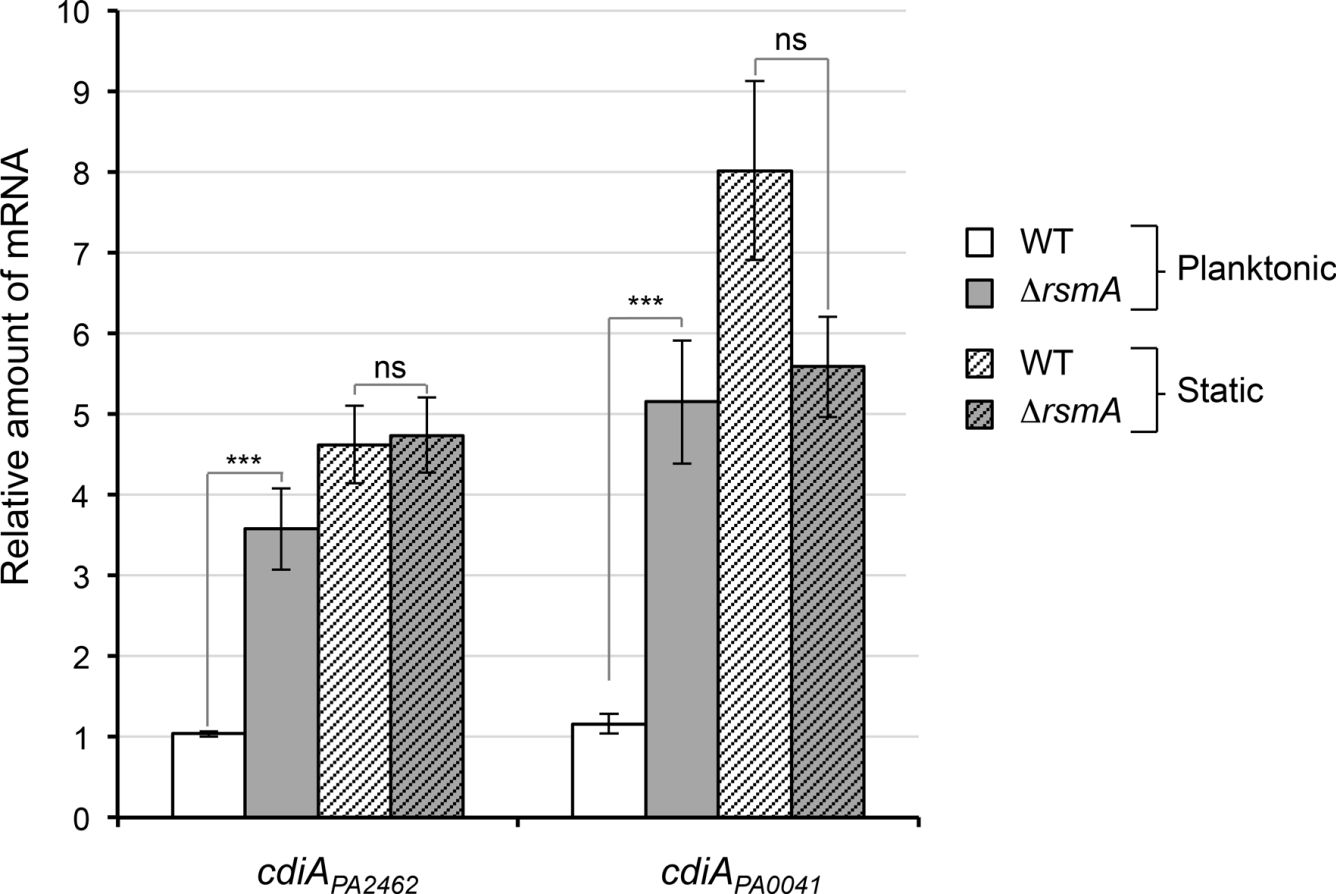
Analysis of *cdiA*_*PA2462*_ and *cdiA*_*PA0041*_ gene expression profile. Expression of *cdiA*_*PA2462*_ and *cdiA*_*PA0041*_ in WT and Δ*rsmA* strains grown 7 h under agitation (planktonic) or without agitation (static) in LB medium. For each gene, expression was normalized to *16S* expression and is shown relative to the WT planktonic condition level. Error bars represent the standard error of the mean from three independent experiments. Data were analyzed for significance using a two-tailed Student’s t-test. ** 0.025<*p*-value <0.01; *** *p*-value<0.01; ns: non-significant.

A genome-wide study showed that the *cdiA*_*PA0041*_ mRNA level of *P*. *aeruginosa* PAK strain increases in Δ*rsmA* (regulator of secondary metabolism) cells [15]. In order to determine whether RsmA influences *cdiA* mRNA levels in *P*. *aeruginosa* PAO1, we performed qRT-PCR in a Δ*rsmA* strain. The deletion of *rsmA* resulted in a ~3.5- to 5-fold increase level of both *cdiA* mRNAs indicating that RsmA acts as a negative regulator (Fig 1). RsmA is a post-transcriptional regulator that blocks the interaction between the ribosome-binding site (RBS) and the 30S ribosomal subunit and subsequently prevents the initiation of translation leading eventually to destabilization and degradation of the transcript [16]. As translation stabilizes mRNA and can affect the accumulation of transcript in cells, the difference in mRNA levels observed between wild-type and Δ*rsmA* could reflect a difference in translation levels. Notably putative RsmA binding sequence overlaps with *cdiA*_*PA0041*_ and *cdiA*_*PA2462*_ RBS indicating that CdiA_PA0041_ and CdiA_PA2462_ are probably directly regulated by RsmA at post-transcriptional level. Interestingly, RsmA downregulates the expression of several genes involved in biofilm development [16]. To investigate whether growth conditions impact the regulation of *cdiA* genes, we compared the level of mRNAs extracted from cells grown under static (in LB medium without agitation) and planktonic conditions. In wild-type cells, qRT-PCR analyses showed that *cdiA*_*PA2462*_ and *cdiA*_*PA0041*_ mRNA levels increase by a 4.5- to 8-fold in static conditions compared to planktonic conditions (Fig 1). Interestingly, in static conditions, we observed that *rsmA* deletion has no longer an effect on transcript levels as mRNA quantities were almost equal in wild-type and Δ*rsmA* cells (Fig 1).

### Wild-type cells inhibit the growth of strains lacking the entire *cdi*_*PA2462*_ or *cdi*_*PA0041*_ loci

**Fig 2:**
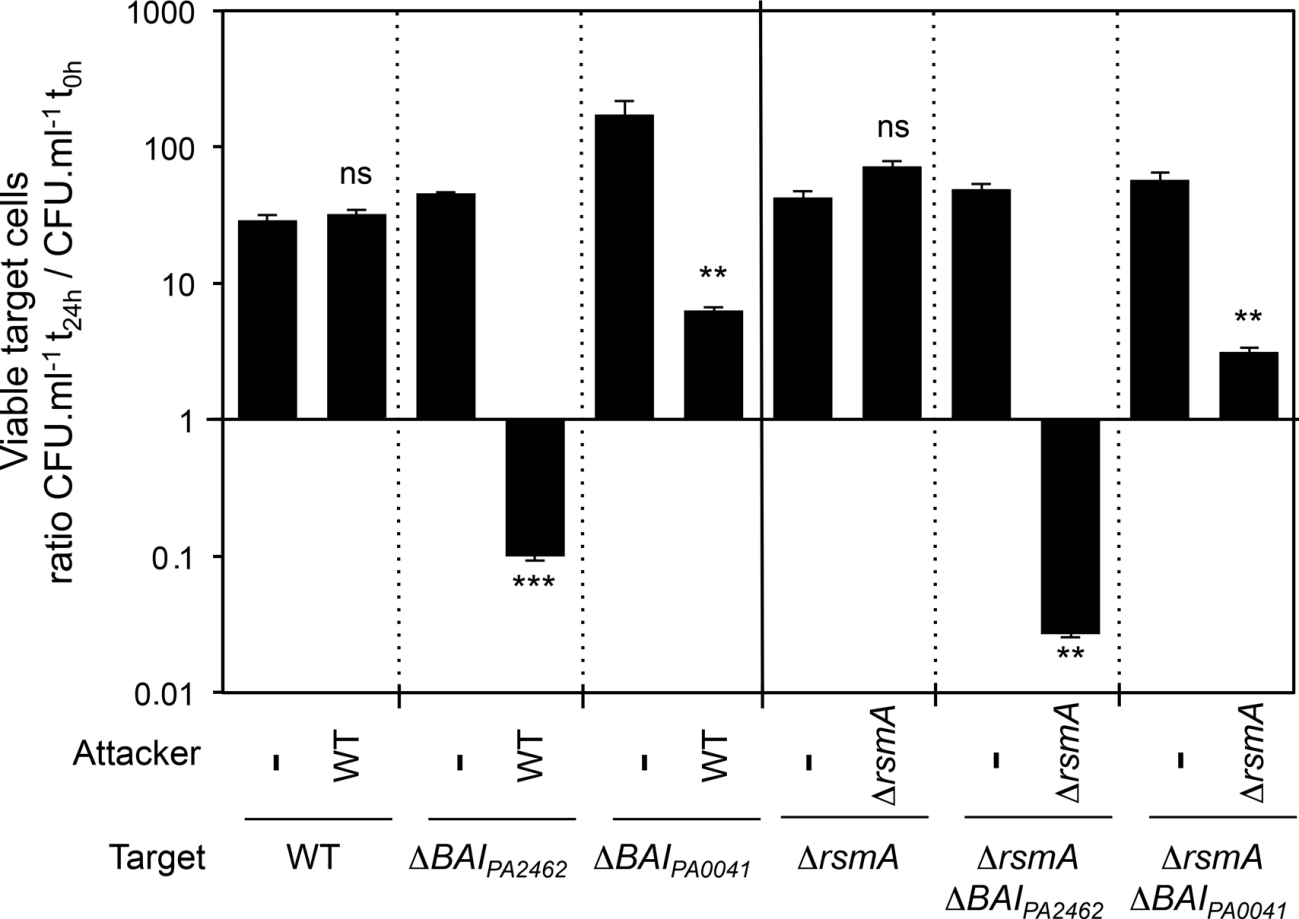
The growth of *cdiBAI*_*PA2462*_ and *cdiBAI*_*PA0041*_ mutants is inhibited by contact with a wild-type strain. Gentamycin-resistant wild-type and mutant target bacteria were mixed with or without wild-type or Δ*rsmA* attackers and the number of viable target cells was calculated as the number of CFU/ml after contact (t_24h_) divided by the number of CFU/ml before contact (t_0h_). Error bars represent the standard error of the mean from three independent experiments.

To assess the role of *cdi*_*PA2462*_ and *cdi*_*PA0041*_ loci in bacterial growth inhibition, we performed competition assays. Attacker and gentamycin-resistant target cells were grown separately 4 h in static condition prior to being mixed at an attacker/target ratio of 4:1. Interestingly, no competition occurred with agitated mixed cultures (data not shown). At the beginning of the experiment (t_0_) and after 24 h, mixed bacteria were serially diluted and plated on gentamycin to determine the CFU/ml of the target. A wild-type target had no growth inhibition when mixed with a wild-type attacker (Fig 2). By contrast, the growth of targets lacking the entire *cdiBAI*_*PA2462*_ or *cdiBAI*_*PA0041*_ locus was inhibited after contact with a wild-type attacker suggesting that at least one gene present in each locus protects from the attack of the wild-type strain (Fig 2). Since our qRT-PCR experiments showed that RsmA is a negative regulator of *cdiA*_*PA2462*_ and *cdiA*_*PA0041*_, we next carried out the competition assays in a Δ*rsmA* background. In this context, both Δ*rsmA*Δ*cdiBAI*_*PA2462*_ and Δ*rsmA*Δ*cdiBAI*_*PA0041*_ mutant targets still showed a growth defect when mixed with a Δ*rsmA* attacker (Fig 2). Although the *rsmA* mutation does not increase the *cdiA*_*PA2462*_ and *cdiA*_*PA0041*_ mRNA levels in static condition (Fig 1), we noticed a larger extent of growth inhibition in the Δ*rsmA* background (Fig 2). Consequently, as growth inhibition was reproducibly higher in the Δ*rsmA* background, only results obtained in this condition are shown below.

### CdiA_PA2462_ and CdiA_PA0041_ are responsible for the growth inhibition phenotype

**Fig 3:**
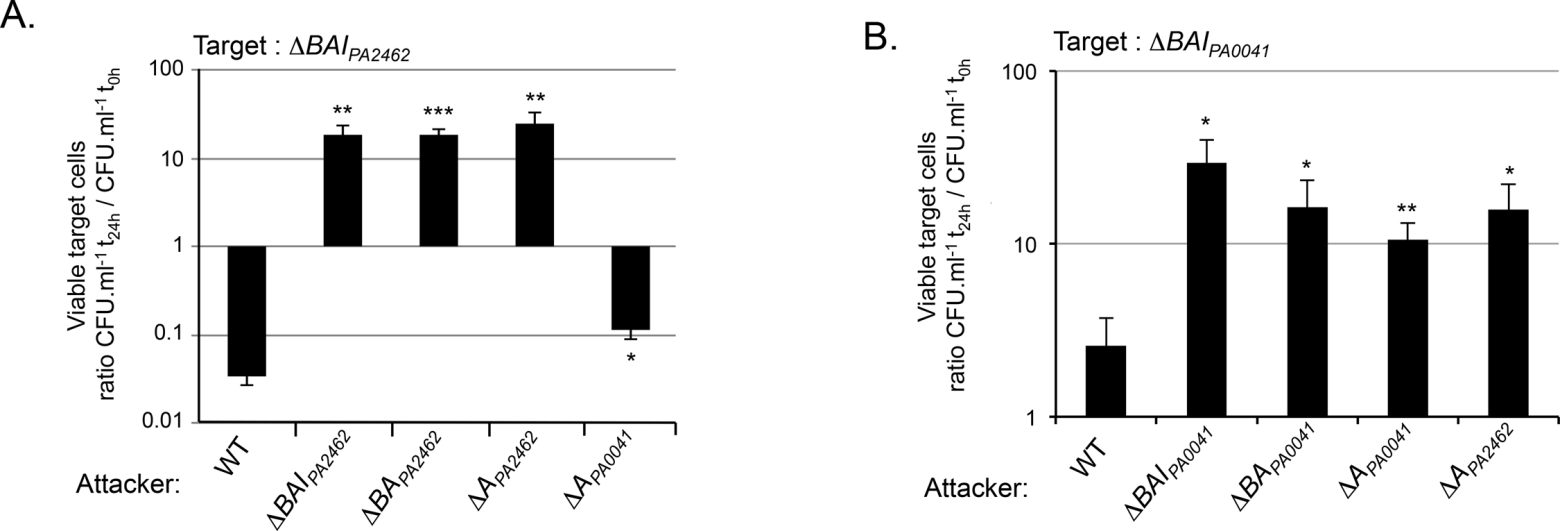
Roles of CdiA_PA2462_, CdiA_PA0041_ in growth competition. All experiments were performed in a Δ*rsmA* background. A) Δ*BAI*_*PA2462*_ and B) Δ*BAI*_*PA0041*_ target strains were mixed with different attacker cells. Error bars represent the standard error of the mean from three independent experiments.

**Fig 4:**
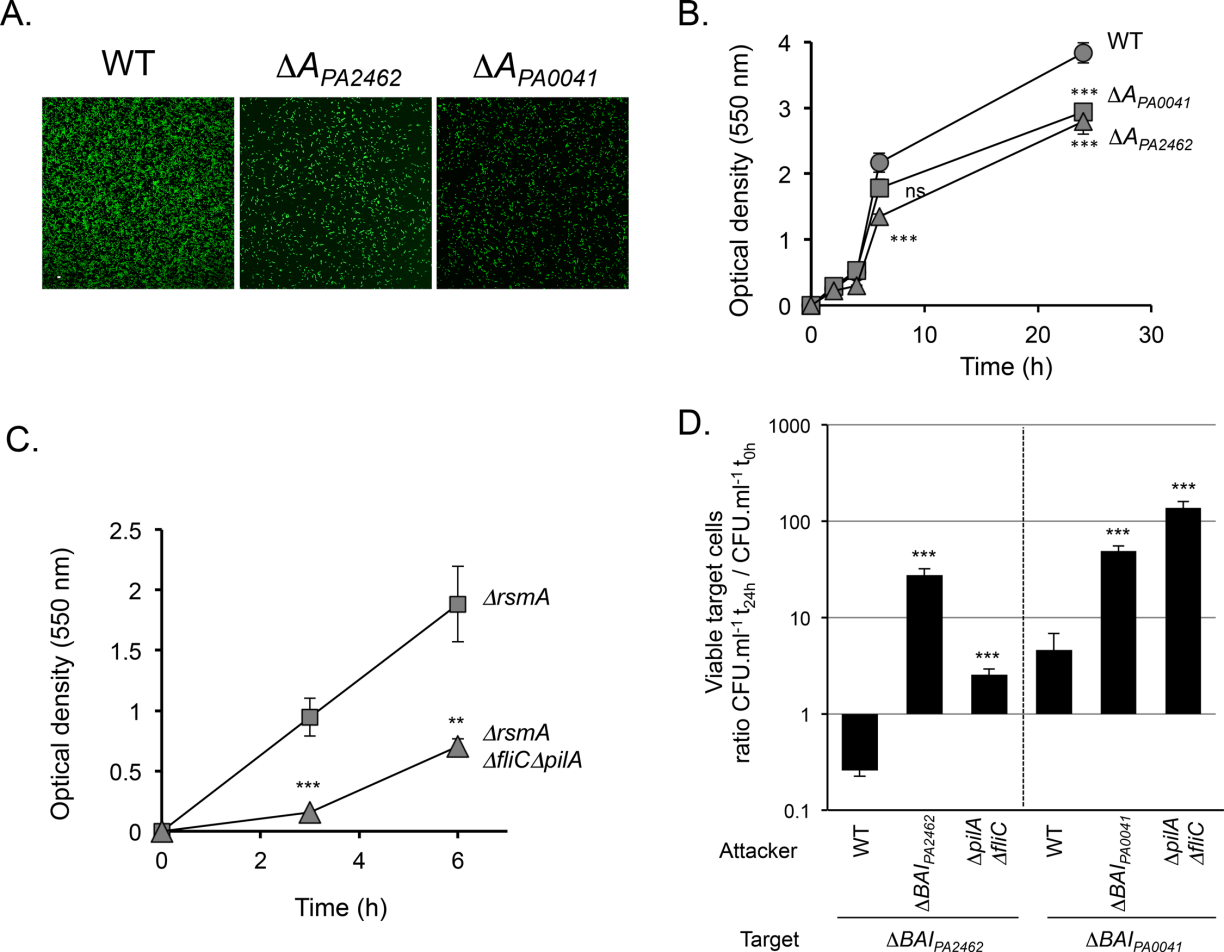
Roles of adhesion and biofilm formation in competition assays. (A). CLSM of adhesive structures formed by WT, *cdiA*_*PA2462*_ and *cdiA*_*PA0041*_ mutants (Δ*A*_*PA2462*_ and Δ*A*_*PA0041*_ respectively) at 8 h post-inoculation. All strains carry a *gfp* gene inserted at the *glmS* locus on the *P*. *aeruginosa* PAO1 chromosome. White scale bar = 10 μm. (B) and (C). After staining with crystal violet, biofilm was quantified over time by measuring the optical density at 550 nm (OD_550_). Each strain was tested in triplicate experiment. Data were examined for significance using a two-tailed Student’s t-test. *** *p*-value<0.01; ns: non-significant. D. Competition experiments were performed in a Δ*rsmA* background. WT or *ΔpilAΔfliC* attacker strains were mixed with Δ*BAI*_*PA2462*_ or *BAI*_*PA0041*_ target bacteria. Error bars represent the standard error of the mean from three independent experiments.

In order to identify the growth inhibition factor, we next generated deletions in the attacker strain genome. Inter-bacterial competition assays showed that attacker strains lacking the entire *cdiBAI*_*PA2462*_ locus, *cdiBA*_*PA2462*_ or only *cdiA*_*PA2462*_ did not inhibit the growth of a Δ*BAI*_*PA2462*_ target strain anymore (Fig 3A). By contrast, a Δ*A*_*PA0041*_ attacker still inhibited Δ*BAI*_*PA2462*_ target cells, although it should be noted that the level of inhibition was lower than what we observed with a wild-type attacker (Fig 3A). We concluded that CdiA_PA2462_ is the main protein able to mediate growth inhibition of Δ*BAI*_*PA2462*_ mutant cells. Similarly, deletion of the entire *cdiBAI*_*PA0041*_ locus, *cdiBA*_*PA0041*_ or *cdiA*_*PA0041*_ genes in an attacker cell altered its capacity to inhibit a Δ*BAI*_*PA0041*_ strain (Fig 3B). Interestingly, similarly to what we observed with Δ*A*_*PA0041*_, the Δ*A*_*PA2462*_ strain did not impede growth of Δ*BAI*_*PA0041*_ target bacteria (Fig 3B). As contact between bacteria is important to observe CDI activity [3], we hypothesized that a defect in adhesion between the Δ*A*_*PA2462*_ attacker and target bacteria might explain the absence of growth inhibition (Fig 3B). To study cell attachment, we visualized bacteria grown in static chambers using confocal laser-scanning microscopy (CLSM) (Fig 4A). Importantly, we first verified that *cdiA* genes were expressed under the minimal media growth conditions used in this assay (S3 Fig). After 8 h, wild-type bacteria attached as a sparse monolayer onto the coverslip. By contrast, *cdiA*_*PA2462*_ or *cdiA*_*PA0041*_ mutants adhered significantly less than wild-type cells (Fig 4A). A growth defect could not explain this decrease of adhesion since the wild-type, *cdiA*_*PA2462*_ and *cdiA*_*PA0041*_ mutant strains showed identical growth curves (data not shown). Next, to determine whether CdiA proteins also play a role in biofilm formation, we quantified biofilm biomass in 96-well polystyrene plates over a 24 h time period. Both *cdiA* mutants generated significantly less biofilm mass than the wild-type strain after 24 h of culture (Fig 4B), showing that CdiA_PA2462_ and CdiA_PA0041_ contribute to the formation of biofilm. The fact that Δ*A*_*PA2462*_ is defective in adhesion and biofilm formation (Fig 4A and 4B), could explain the incapacity of Δ*A*_*PA2462*_ to inhibit Δ*BAI*_*PA0041*_ growth (Fig 3B), suggesting that adhesive activities are essential for inhibiting Δ*BAI*_*PA0041*_. By contrast, although Δ*A*_*PA0041*_ is impaired in process of adhesion (Fig 4A and 4B), it still retained an inhibiting activity against Δ*BAI*_*PA2462*_ target strains (Fig 3A), implying that an adhesion defect does not interfere with the capacity to inhibit the growth Δ*BAI*_*PA2462*_ cells. To strengthen our hypotheses, we tested a mutant with defects in cell adhesion/biofilm formation speculating that it would display similar competition defects. We used an attacker mutant devoid of type IVa pili and of flagella (Δ*pilA*Δ*fliC*) and confirmed that it displayed a defect in biofilm formation (Fig 4C). Competition assays showed that the Δ*pilA*Δ*fliC* mutant still inhibited the growth of Δ*BAI*_*PA2462*_ target bacteria but to a lesser extent than a wild-type attacker (Fig 4D). By contrast, no growth defect of Δ*BAI*_*PA0041*_ target cells was observed in contact with Δ*pilA*Δ*fliC* mutant (Fig 4D). Altogether these results confirmed that adhesion process seems essential to the inhibition of Δ*BAI*_*PA0041*_ cells and that Δ*BAI*_*PA2462*_ bacteria are less sensitive to a cell adhesion/biofilm formation defect.

### Identification of potential immunity proteins: roles of CdiI_PA2462_ and CdiI_PA0041_

**Fig 5:**
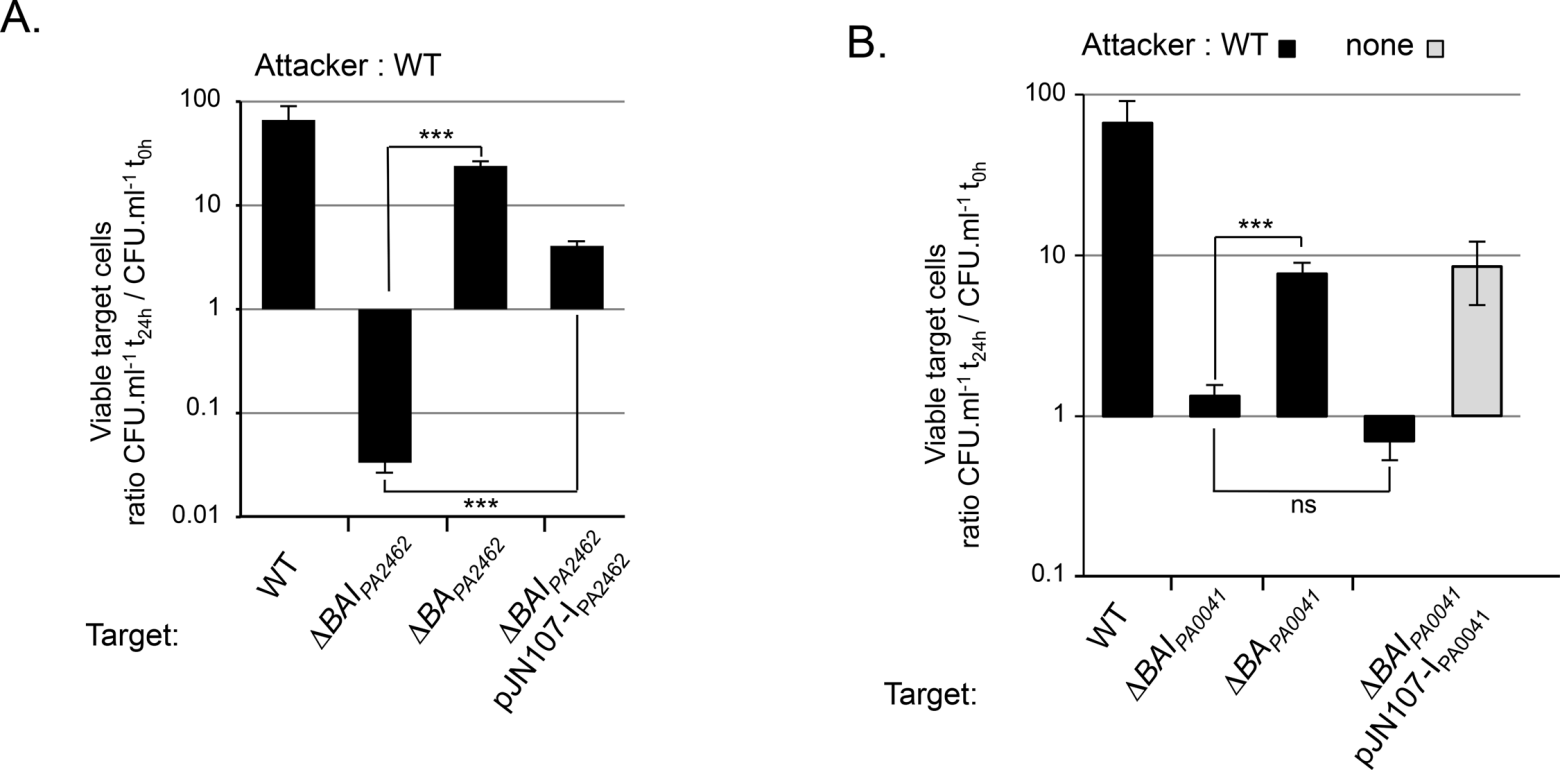
CdiI_PA2462_ and CdiI_PA0041_ play a protective role. All experiments were carried out in a Δ*rsmA* background. Wild-type (WT) attacker was mixed with different gentamycin-resistant mutant targets: (A) Δ*BAI*_*PA2462*_ carrying or not the pJN107 plasmid encoding the CdiI_PA2462_ protein or the *BA*_*PA2462*_ mutant. No arabinose was added to produce CdiI_PA2462_ from pJN107-I_PA2462_; (B) *BAI*_*PA0041*_ carrying or not the pJN107 plasmid encoding the CdiI_PA0041_ protein or the *BA*_*PA0041*_ mutant. As control, Δ*BAI*_*PA0041*_ expressing *cdiI*_*PA0041*_ was tested alone in inter-bacterial competition (grey bar). 0.5% arabinose was used to induce CdiI_PA0041_.

To determine whether the *cdiI* genes encode immunity proteins, we performed competition experiments with target cells that only produce CdiI proteins. As shown in Fig 5A, wild-type bacteria inhibit the growth of a Δ*BAI*_*PA2462*_ (CdiI^*-*^) target strain but not the one of Δ*BA*_*PA2462*_ (CdiI^*+*^) strain or a Δ*BAI*_*PA2462*_ strain producing the CdiI_PA2462_ protein in *trans*. These results show that wild-type cells can only inhibit bacteria that do not express *cdiI*_*PA2462*_ and hence suggest that CdiI_PA2462_ alone protects from the attack. Similarly, a Δ*BA*_*PA0041*_ (CdiI^*+*^) target is unaffected by the growth inhibitory activity of a wild-type strain. However, a Δ*BAI*_*PA0041*_ strain containing a plasmid carrying *cdiI*_*PA0041*_ remains sensitive to a wild-type attack (Fig 5B). This sensitivity could be due to a growth defect associated with CdiI_PA0041_ production or a lack of *cdiI*_*PA0041*_ expression. We ruled out the first hypothesis as the growth of Δ*BAI*_*PA0041*_ cells producing CdiI_PA0041_ is not affected in absence of attacker strains (Fig 5B, grey bar) and propose that the observed sensitivity comes from a defect in *cdiI*_*PA0041*_ expression. Altogether, these data indicate that a wild-type strain inhibits the growth of *cdiI*_*PA0041*_ mutant cells but do not directly prove that the CdiI_PA0041_ is an immunity protein.

### CdiA-CT_PA2462_/CdiI_PA2462_ and CdiA-CT_PA0041_/CdiI_PA0041_ encode specific toxin/immunity pairs

**Fig 6:**
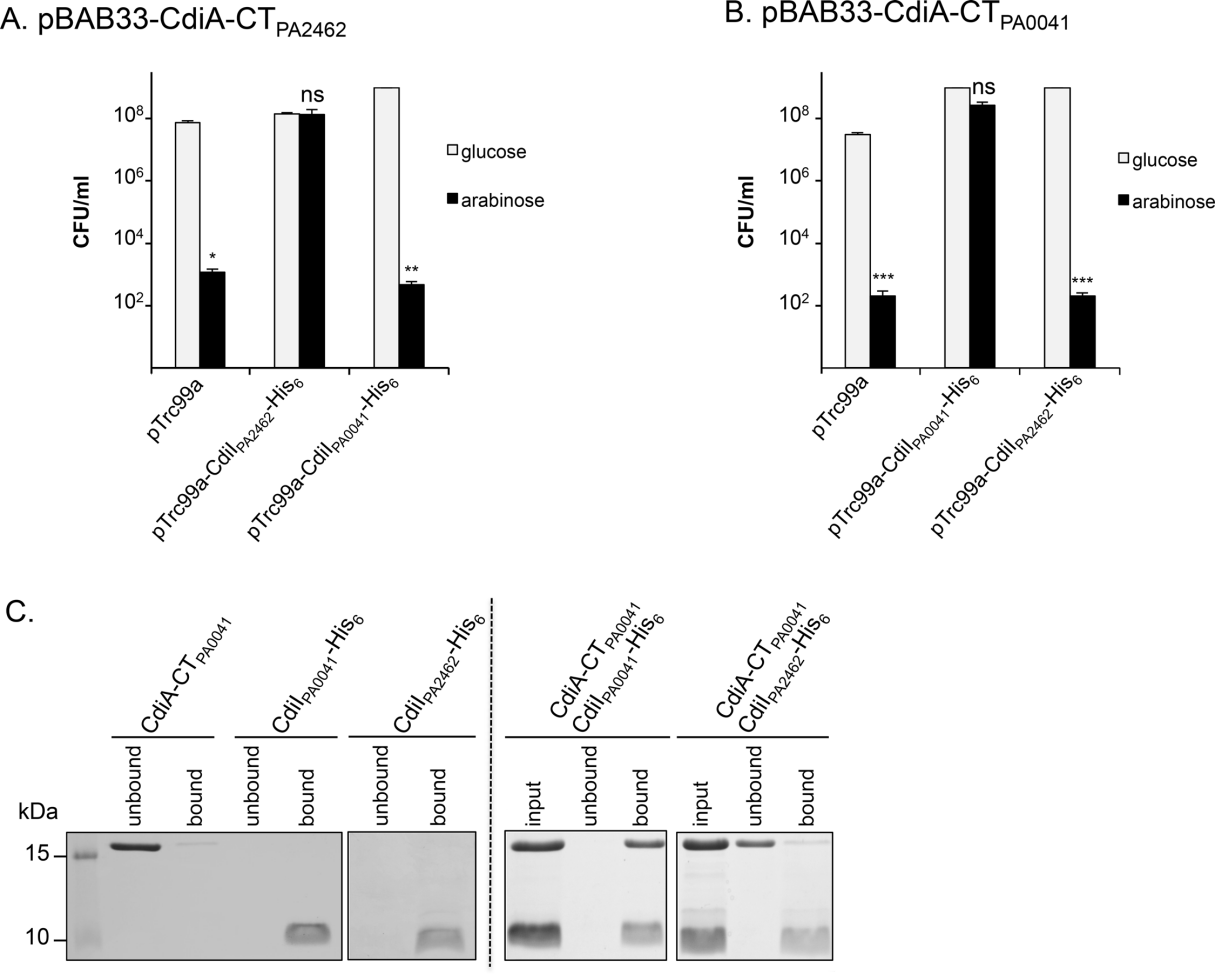
Intracellular toxicity of CdiA-CT_PA2462_ and CdiA-CT_PA0041_ in *E*. *coli* cells and protection by their cognate CdiI immunity proteins. Production of (A) CdiA-CT_PA2462_ and (B) CdiA-CT_PA0041_ were repressed with 0.5% glucose (grey bars) or induced with 1% arabinose (black bars). Error bars represent the standard error of the mean from three independent experiments. C. Purified CdiA-CT_PA0041_ and CdiI-His_6_ proteins were mixed *in vitro* with Ni^2+^-NTA resin. Fractions were analyzed by SDS-PAGE and Coomassie blue staining. Control experiments with purified proteins incubated alone are shown on the left panel while CdiA-CT/CdiI-His_6_ mixtures are shown on the right panel. Marker lane (kDa) is shown on the left.

CT domains of CdiA_PA2462_ and CdiA_PA0041_ show homologies with nuclease domains. To assess their putative toxic function, we cloned the DNA fragments encoding both CT-domains under the arabinose inducible P_*BAD*_ promoter in *E*. *coli* using pBAD33. While we readily cloned *cdiA-CT*_*PA0041*_, we only obtained one clone with *cdiA-CT*_*PA2462*_ albeit with a deletion in the Shine Dalgarno sequence likely decreasing the production of the CT domain (S1 Table). This result strongly suggests that the production of CdiA-CT_PA2462_ in the cytoplasm is toxic. In presence of arabinose, the number of CFU/ml of *E*. *coli* strain producing either CdiA-CT_PA2462_ or CdiA-CT_PA0041_ decreased drastically by 5 and 6 logs respectively (Fig 6A and 6B). Thus, the production of these domains in *E*. *coli* is toxic.

CdiI_PA2462_ and potentially CdiI_PA0041_ protect from *P*. *aeruginosa* PAO1 attack, raising the possibility that these proteins would protect *E*. *coli* from the toxicity of the CT-domains. In order to test this hypothesis, the *cdiI*_*PA2462*_ and *cdiI*_*PA0041*_ genes were fused to a 6xHis epitope-encoding sequence downstream the IPTG-inducible P_*trc*_ promoter. The production of CdiI_PA2462_-His_6_ and CdiI_PA0041_-His_6_ protected *E*. *coli* from CdiA-CT_PA2462_ and CdiA-CT_PA0041_ toxicity respectively as CFU/ml counts with arabinose and glucose were comparable (Fig 6A and 6B). By contrast, the number of CFU/ml of *E*. *coli* co-producing CdiA-CT_PA2462_ with CdiI_PA0041_-His_6_ or CdiA-CT_PA0041_ with CdiI_PA2462_-His_6_ decreased by 5 and 6 logs respectively, showing that these systems are not interchangeable. The inability to rescue *E*. *coli* growth was not due to the absence of CdiI production as shown by western-blot analyses (S4A and S4B Fig). Altogether, these results demonstrate that CdiA-CT_PA2462_/CdiI_PA2462_ and CdiA-CT_PA0041_/CdiI_PA0041_ function as toxin/immunity systems.

We speculated that the CdiI proteins prevent the toxicity of CdiA-CT through specific binding inhibition. To test pair-wise interaction, the CdiI and CdiA proteins were purified and their interactions assessed using Ni^2+^-affinity pull-down experiments. While the CdiI_PA2462_-His_6_, CdiI_PA0041_-His_6_ and CdiA-CT_PA0041_ proteins were readily produced and purified, we failed to clone a non-mutated *cdiA*-*CT*_*PA2462*_-endoding DNA even in the presence of the cognate *cdiI*_*PA2462*_ gene, presumably due to high toxicity of CdiA-CT_PA2462_. Therefore, only the CdiI_PA0041_/CdiA-CT_PA0041_ interaction was assayed. CdiI_PA0041_-His_6_ but not CdiI_PA2462_-His_6_ specifically retained CdiA-CT_PA0041_ (Fig 6C) showing that CT-domain of CdiA_PA0041_ interacts specifically to its cognate CdiI_PA0041_ immunity protein *in vitro*.

#### CDI systems are widely distributed among *Pseudomonas* species

To identify other potential *Pseudomonas* CDI-encoding loci, we performed a BLAST search using the CdiA_PA2462_ and CdiA_PA0041_ proteins from *P*. *aeruginosa* PAO1 and analyzed the sequences flanking each of the identified *cdiA* homologues. We considered as part of a potential CDI locus (listed in Table 1) *cdiA* homologues identified immediately downstream of a *cdiB* homologue and upstream of a small non-annotated or unknown ORF.

**Table 1:** Predicted *Pseudomonas* CDI-encoding locus.

Strain	CdiB	CdiA / CT domain^a^	motif	CdiI*	Stop-Start /lenght^b^	Class
*P*. *syringae* pv.tomato DC3000	PSPTO_3230	PSPTO_3229	PT-HINT	CdiI- PSPTO_3229*	**TAG**CTATG /132	I
*P*. *fluorescens* A506	PflA506_0158	PflA506_0159	PT-HINT/DUF637	PflA506_0160		I
*P*. *fluorescens* SBW25	PFLU_3192	PFLU_3191 / Tox-REase-7 (PF15649)	PT-HINT	PFLU_3190		I
*P*. *protegens* Pf-5	PFL_1551	PFL_1552	PT-HINT/DUF637	CdiI-PFL_1552*	**TGA**TTTATG /133	I
*P*. *fluorescens* SBW25	PFLU_0148	PFLU_0148	PT-VENN	CdiI-PFLU_0148*	ATGAAAAAG**TGA** /72	II
*P*. *fluorescens* SBW25	PFLU_3246	PFLU_3246 / Tox-HNH-HHH (PF15637)	PT-VENN	CdiI-PFLU_3246*	**TA****A**TG /143	II
*P*. *aeruginosa* NCGM2.S1	NCGM2_0040	NCGM2_0041 / MafB19-deam (PF14437)	PT-VENN	CdiI-NCGM2_0041*	AATA**TG****A** /133	II
*P*. *fulva* 12-X	Psefu_0120	Psefu_0121/ Tox-REase-7 (PF15649)	none	Psefu_0122		III
*P*. *fluorescens* F113	PSF113_0792	PSF113_0793 / AHH (PF14412)	none	PSF113_0794		III
*P*. *fluorescens* A506	PflA506_2790	PflA506_2789	none	CdiI-PflA506_2789*	**TAG**AGAAATTTTATATG /68	III
*P*. *entomophila* L48	PSEEN_3947	PSEEN_3946	DUF637	CdiI-PSEEN_3946*	GCAG**TG****A**A /129	IV
*P*. *brassicacearum* DF41	CD58_03745	CD58_03750 / Ribonuclease/ribotoxin (IPR016191)	DUF637	CD58_03755		IV
*P*. *protegens* CHA0	PFLCHA0_c15900	PFLCHA0_c15910	DUF637	CdiI-PFLCHA0_c15910*	**TAG**AGGAGGACGGTTATG /160	IV
*P*. *aeruginosa* PAO1	PA2463	PA2462 /RNAse EndoU fold (PF14436)	none	CdiI- PA2462*	**TAG**GTCTTTTATG /84	V
*P*. *aeruginosa* PA14	PA14_32780	PA14_32790	DUF637	CdiI-PA14_32790*	AG**TG****A**A /113	V
*P*. *aeruginosa* PA7	PSPA7_2776	PSPA7_2777 / CDI_toxin_Bp1026b_like	DUF637	CdiI- PSPA7_2777*	GA**TG****A**GC /130	V
*P*. *aeruginosa* LESB58	PALES_2831	PALES_28341 / Toxin deaminase (PF14424)	none	CdiI-PALES_28341*	G**TA****A**TGG /117	V
*P*. *aeruginosa* PAO1	PA0040	PA0041 / CDI_toxin_Bp1026b_like	DUF637	CdiI-PA0041*	C**TGA**TTATG /84	V
*P*. *aeruginosa* PA7	PSPA7_0042	PSPA7_0044	DUF637	PSPA7_0045		V
*P*. *aeruginosa* LESB58	PALES_00391	PALES_00401	DUF637	CdiI- PALES_00401*	AAAA**TG****A**TT /106	V
*P*. *aeruginosa* PACS2	PaerPA_01000042	PaerPA_01000043 /Toxin_49 (PF15529)	DUF637	CdiI- PaerPA_01000043*	CA**TG****A**A /156	V
*P*. *aeruginosa* PA14	PA14_00490	PA14_00510	DUF637	CdiI-PA14_00510*	C**TG****A**TGA/155	V
*P*. *aeruginosa* NCGM2.S1	NCGM2_3465	NCGM2_3464	DUF637	CdiI- NCGM2_3464*	**TGA**GTTAATATG /106	V
*P*. *aeruginosa* DK2	PADK2_00205	PADK2_00210 / Pyocin large subunit (COG5529)	DUF637	CdiI-PADK2_00210*	**TAA**GGAGAGCCCAATG /80	V
*P*. *syringae* pv.tomato DC3000	PSPTO_3230	PSPTO_3229	PT-HINT	CdiI- PSPTO_3229*	**TAG**CTATG /132	
*P*. *fluorescens* A506	PflA506_0158	PflA506_0159	PT-HINT/DUF637	PflA506_0160		
*P*. *fluorescens* SBW25	PFLU_3192	PFLU_3191 / Tox-REase-7 (PF15649)	PT-HINT	PFLU_3190		
*P*. *protegens* Pf-5	PFL_1551	PFL_1552	PT-HINT/DUF637	CdiI-PFL_1552*	**TGA**TTTATG /133	
*P*. *fluorescens* SBW25	PFLU_0148	PFLU_0148	PT-VENN	CdiI-PFLU_0148*	ATGAAAAAG**TGA** /72	
*P*. *fluorescens* SBW25	PFLU_3246	PFLU_3246 / Tox-HNH-HHH (PF15637)	PT-VENN	CdiI-PFLU_3246*	**TA****A**TG /143	
*P*. *aeruginosa* NCGM2.S1	NCGM2_0040	NCGM2_0041 / MafB19-deam (PF14437)	PT-VENN	CdiI-NCGM2_0041*	AATA**TG****A** /133	
*P*. *fulva* 12-X	Psefu_0120	Psefu_0121/ Tox-REase-7 (PF15649)	none	Psefu_0122		
*P*. *fluorescens* F113	PSF113_0792	PSF113_0793 / AHH (PF14412)	none	PSF113_0794		
*P*. *fluorescens* A506	PflA506_2790	PflA506_2789	none	CdiI-PflA506_2789*	**TAG**AGAAATTTTATATG /68	
*P*. *entomophila* L48	PSEEN_3947	PSEEN_3946	DUF637	CdiI-PSEEN_3946*	GCAG**TG****A**A /129	
*P*. *brassicacearum* DF41	CD58_03745	CD58_03750 / Ribonuclease/ribotoxin (IPR016191)	DUF637	CD58_03755		
*P*. *protegens* CHA0	PFLCHA0_c15900	PFLCHA0_c15910	DUF637	CdiI-PFLCHA0_c15910*	**TAG**AGGAGGACGGTTATG /160	
*P*. *aeruginosa* PAO1	PA2463	PA2462 /RNAse EndoU fold (PF14436)	none	CdiI- PA2462*	**TAG**GTCTTTTATG /84	
*P*. *aeruginosa* PA14	PA14_32780	PA14_32790	DUF637	CdiI-PA14_32790*	AG**TG****A**A /113	
*P*. *aeruginosa* PA7	PSPA7_2776	PSPA7_2777 / CDI_toxin_Bp1026b_like	DUF637	CdiI- PSPA7_2777*	GA**TG****A**GC /130	
*P*. *aeruginosa* LESB58	PALES_2831	PALES_28341 / Toxin deaminase (PF14424)	none	CdiI-PALES_28341*	G**TA****A**TGG /117	
*P*. *aeruginosa* PAO1	PA0040	PA0041 / CDI_toxin_Bp1026b_like	DUF637	CdiI-PA0041*	C**TGA**TTATG /84	
*P*. *aeruginosa* PA7	PSPA7_0042	PSPA7_0044	DUF637	PSPA7_0045		
*P*. *aeruginosa* LESB58	PALES_00391	PALES_00401	DUF637	CdiI- PALES_00401*	AAAA**TG****A**TT /106	
*P*. *aeruginosa* PACS2	PaerPA_01000042	PaerPA_01000043 /Toxin_49 (PF15529)	DUF637	CdiI- PaerPA_01000043*	CA**TG****A**A /156	
*P*. *aeruginosa* PA14	PA14_00490	PA14_00510	DUF637	CdiI-PA14_00510*	C**TG****A**TGA/155	
*P*. *aeruginosa* NCGM2.S1	NCGM2_3465	NCGM2_3464	DUF637	CdiI- NCGM2_3464*	**TGA**GTTAATATG /106	
*P*. *aeruginosa* DK2	PADK2_00205	PADK2_00210 / Pyocin large subunit (COG5529)	DUF637	CdiI-PADK2_00210*	**TAA**GGAGAGCCCAATG /80	

^a^ CT domain: identified conserved domains using Interproscan5 program and NCBI database

^b^ stop codon of *cdiA* gene and start codon of *cdiI* gene are in bold and underlined respectively, number indicate the length of the predicted CdiI protein

*: non-annotated ORF encoding potential CdiI proteins

**Fig 7:**
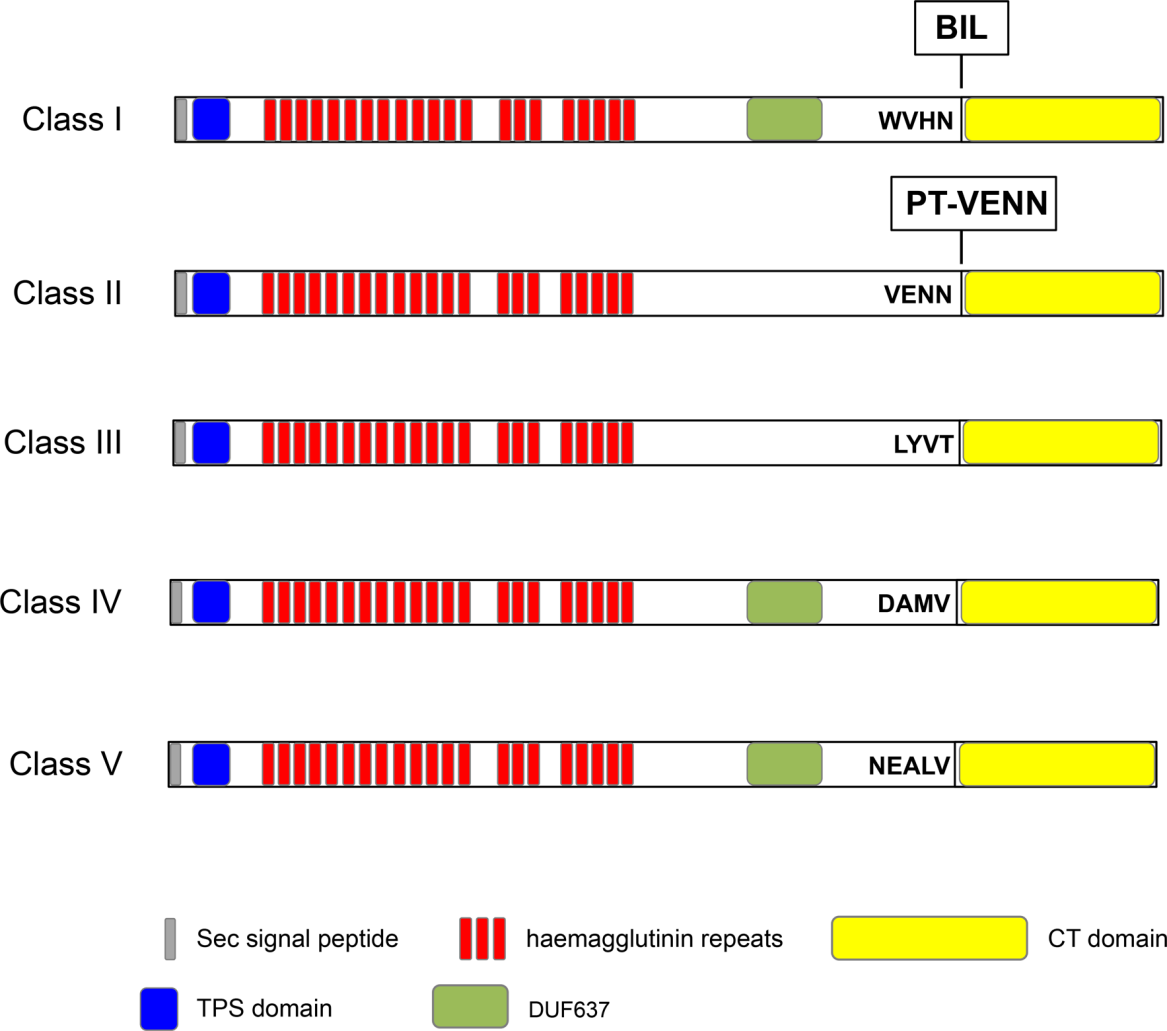
Representative CdiA proteins of each class of pre-toxin motif. The signal peptide (SP) is a sequence potentially recognized by the sec machinery and cleaved during the export through the inner membrane. TPS domain found in N-terminal part of TpsA allows targeting to the TpsB transporter and subsequent translocation through the outer membrane. Haemagglutinin repeats are highly divergent repeats found in FHA-like proteins. DUF637 is a conserved region found in bacterial haemagglutinins or hemolysins. WVHN, VENN, LYVT, DAMV, NEALV are the pre-toxin motifs delimiting the conserved N-terminal and the variable CT domains.

Interestingly, we identified CDI systems in *P*. *aeruginosa* species, generally found in human infection, but also in *Pseudomonas* species associated with plants (Table 1). While *P*. *protegens*, *P*. *fluorescens* and *P*. *brassicacearum* are plant-protecting bacteria, others, such as *P*. *syringae*, are phytopathogens. All predicted *Pseudomonas* CdiA (*p*CdiA) sequences contain a Sec signal peptide allowing translocation across the inner membrane, a conserved TPS domain recognized by cognate CdiB transporter and repetitive regions with haemagglutinin-type repeats (Fig 7). In addition, all *p*CdiA exhibit a C-terminal variable region (Fig 7, CT domain) with no amino-acid similarities except the CT domains of CdiA_PFLU_3191_ and CdiA_Psefu_0121_ from *P*. *fluorescens* SBW25 and *P*. *fulva* 12-X respectively, sharing 70% identity. Similarly to the high variability of CT domains, the sequences of small ORFs, immediately downstream of *pcdiA*, are also variable, except for PFLU_3191 and Psefu_0122, the predicted immunity proteins of CdiA_PFLU_3191_ and CdiA_Psefu_0121_, which also share 70% identity.

Based on the amino-acid alignment of *p*CdiA proteins and the observed sequence identity between them, we classified the pre-toxin motifs at the transition between the N-termini and CT-variable domains into five distinct classes (Fig 7 and S5 Fig). Proteins with the class I peptide motif contain a bacterial intein-like domain (BIL domain) [17] sandwiched between a conserved motif (WVHN) and the CT variable domain. Class II pre-toxin motif represents the highly conserved VENN motif (PF04829) found in “*E*. *coli*-type” CDI systems [5]. In class III, IV and V pre-toxin motifs, the peptides that separate the N-terminus domain from the variable CT region are LYVT, DAMV and NEVLA respectively. Class I, IV and V exhibit a small-helical DUF637 domain of unknown function (Table 1 and Fig 7). Some CT domains of *p*CdiA proteins carry potential nuclease activities (Table 1) but ~ 50% of the CT domains have no predicted function. Sequence analyses of predicted immunity proteins revealed that CdiI_PSPTO_3229_ and CdiI_PALES_28341_ contains respectively SMI1 and Imm2 domains, typically found in immunity proteins of bacterial polymorphic toxin systems [2,18]. In addition, the CD58_03755 protein encoded by *P*. *brassicacearum* DF41 contains a barstar domain predicted to inhibit specifically the Barnase protein, an extracellular ribonuclease of *Bacillus amyloliquefaciens* [19]. Except for these 3 proteins, no known domains have been found in other predicted immunity proteins.

### *P*. *aeruginosa* PA7, PA14 and *P*. *syringae* pv. tomato DC3000 encode functional toxin/immunity pairs

**Fig 8:**
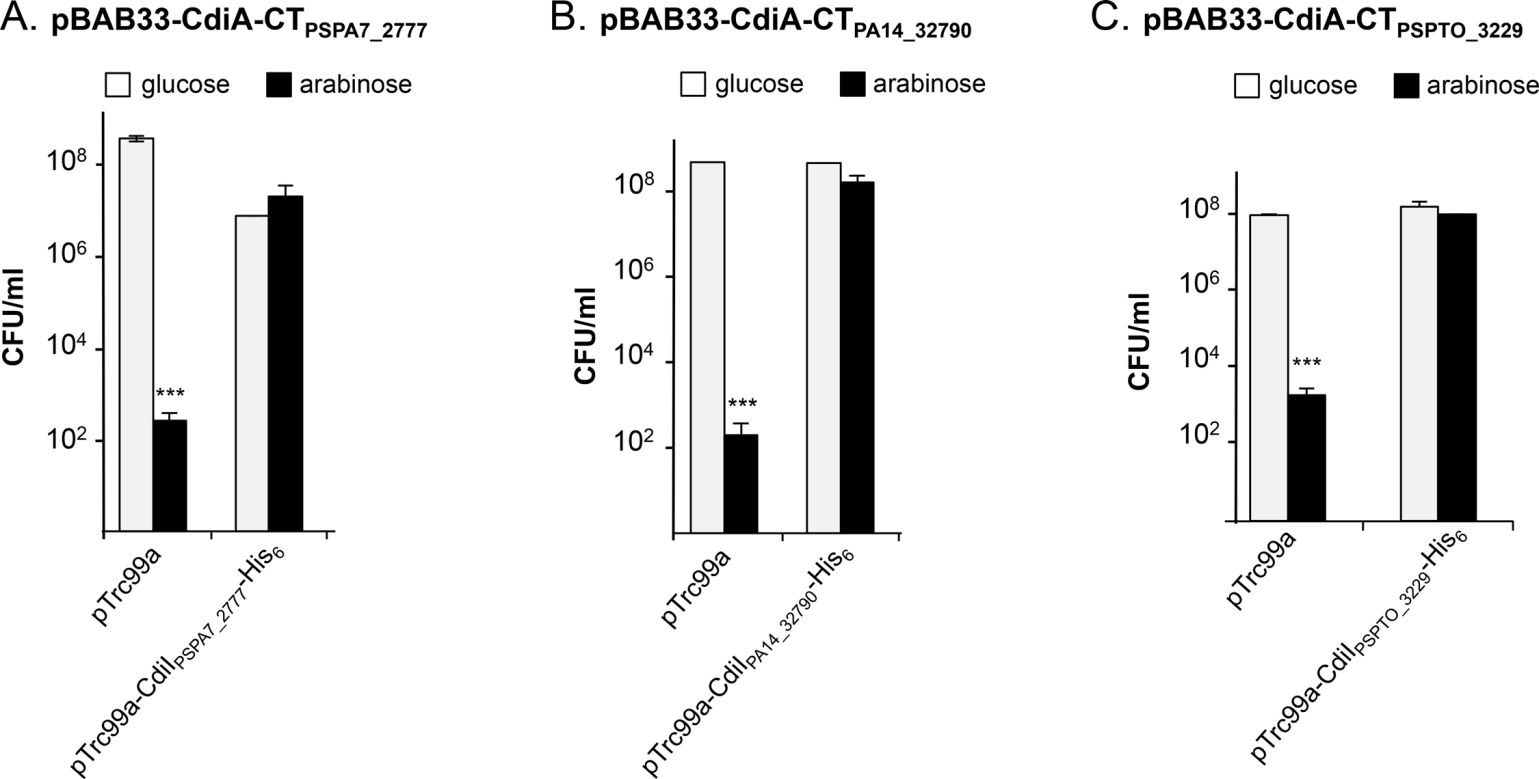
CdiA-CT encoded by *P*. *aeruginosa* PA7, PA14 and *P*. *syringae* pv. tomato DC3000 inhibit *E*. *coli* cell growth. Production of (A) CdiA-CT_PSPA7_2777,_ (B) CdiA-CT_PA14_32790_ and (C) CdiA-CT_PSPTO_3229_ was repressed with 0.5% glucose (grey bars) and induced with 1% arabinose (black bars). CdiI-His_6_ proteins were produced from pTrc99a with 1 mM IPTG. The number of CFU/ml was calculated after 6 h of culture and the graphs show the mean of three independent experiments.

To investigate the functionality of some of the newly discovered CDI toxins (Table 1), we focused on three CdiA-CT domains with predicted or unknown activities (Fig 7). CdiA-CT_PSPA7_2777_ found in *P*. *aeruginosa* PA7 potentially carries nuclease activity based on the homology with the *B*. *pseudomallei* 1026b CT domain (Table 1 and [6]). On the other hand, no predicted functions have been attributed to the CdiA-CT_PA14_32790_ and CdiA-CT_PSPTO_3229_ respectively produced by *P*. *aeruginosa* PA14 and *P*. *syringae* pv. tomato DC3000. To assess putative toxic function, CT-domains were separately produced in *E*. *coli* with or without their cognate immunity proteins. Production of each CT-domains with arabinose significantly inhibited *E*. *coli* cell growth (Fig 8A, 8B and 8C) while co-production of the cognate CdiI protein completely alleviated the cell growth inhibition showing that CdiA-CT_PSPA7_2777_/CdiI_PSPA7_2777_ and CdiA-CT_PA14_32790_/CdiI_PA14_32790_ constitute specific toxin/immunity systems. In addition, these results confirm the predicted toxic activity of CdiA-CT_PSPA7_2777_ and strongly suggest that CdiA-CT_PA14_32790_ and CdiA-CT_PSPTO_3229_ act in the cytoplasm of the target cells.

## Discussion

The results presented demonstrate that *Pseudomonas* species encode CDI systems: (i) CT domains of *Pseudomonas* CdiA proteins are highly variable and toxic when produced intracellularly in *E*. *coli* cells; (ii) *Pseudomonas cdiI* genes encode immunity proteins that neutralize the toxicity in an allele-specific manner; (iii) *Pseudomonas* CdiA function in growth competition and wild-type *P*. *aeruginosa* can outcompete a Δ*BAI* mutant that does not express the cognate immunity gene; and (iv) such as in *Burkholderia* and *E*. *coli* systems [10,11], CdiA proteins contribute to biofilm formation.

In this study we defined four novel classes of peptide motif separating the conserved N-terminal and variable CT regions, in addition to the common characteristics found in *E*. *coli*- and *Burkholderia*-type CDI systems. Indeed, we identified CdiA proteins with class I peptide motif harboring a bacterial intein-like (BIL) domain [17] related to the Hedgehog/Intein (Hint) domain implicated in the maturation of inteins and Hogs proteins [20]. The BIL domain of *P*. *syringae* CdiA_PSPTO_3230_ is active in autoproteolytic cleavage [17] suggesting that CdiA proteins might have the intrinsic ability to auto-cleave before being delivered across the target-cell envelope. Our analyses suggest that the BIL domain is restricted to CdiA encoded by *Pseudomonas* phytopathogens (Table 1). Similarly, class V peptide motif has been identified only in CdiAs encoded by *P*. *aeruginosa* species. It has been suggested that the VENN sequence might be important for homologous recombination in order to acquire new *cdiA-CT/cdiI* pairs by horizontal gene transfer and consequently to participate in the variability of secreted toxins. The diversity of motifs that define the variable CT-region in *Pseudomonas* species could explain the various predicted toxin activities of the CdiA-CT domain (Table 1) but also reflects the high genomic plasticity and capacity of *Pseudomonas* to capture genes from its environment. We identified at least 10 different putative functions for the CT-domains, all of them possibly targeting nucleic acids. However, half of the identified CT-domains do not belong to a defined protein family domain and characterization of their biological functions remains a challenging question.

While the role and mechanisms of delivery of CDI toxins in enterobacteria and *Burkholderia* species are now better understood, the regulatory pathways underlying CDI systems expression remain unknown for most of these systems. Except *E*. *coli* EC93 which constitutively expresses its CDI system [3], bacteria tightly regulate the expression of CDI-encoding genes. For example, *Burkholderia* express *bcp* genes in a stochastic manner, with only a subset of bacteria expressing these genes to high level [12] and *bcp* expression is strongly induced by cell density in cells grown on solid media and reduced in cells grown in liquid [10]. Similarly, *Dickeya dadantii* 3937 and probably *Erwinia chrysanthemi* EC16 activate their CDI systems only when in contact with plant hosts [5]. In this study, we identified RsmA as a negative regulator of the expression of *P*. *aeruginosa* PAO1 CDI systems. In *P*. *aeruginosa*, the activity of RsmA is controlled by a complex regulatory system. This includes the RetS and LadS sensor kinases and the histidine phosphotransfer protein HptB which all converge on the master two-component regulator system GacS/GacA to influence the levels of the RsmY and RsmZ sRNAs, which ultimately modulates the expression of target genes by titrating RsmA [16]. Putative RsmA binding sequences overlap the RBS of *cdiA*_*PA2462*_ and *cdiA*_*PA0041*_ ([15] and our study) suggesting that these genes are directly regulated by RsmA at a post-transcriptional level. Remarkably, all 5’unstranslated region of *P*. *aeruginosa cdiA* genes identified in this study contain identical RsmA boxes suggesting a conserved post-transcriptional regulation by RsmA. However, the contribution of RetS, HptB, LadS and GacS in expression of *cdi* genes remains to be determined. Additional experiments will be necessary to identify the regulatory circuits controlling other *Pseudomonas cdi* gene expression.

In contrast to the CDI-mediated competition that occurs in shaking conditions in *E*. *coli* [3], we observed inter-bacterial competition in *Pseudomonas* only when bacteria were mixed in static conditions, either on solid surface or in liquid. The gene expression patterns cannot explain the disparity observed in competition experiments since the maximum mRNA levels were obtained in *rsmA* mutant grown either in shaking or static conditions (Fig 1). CDI-mediated growth inhibition occurs upon direct cell-cell contact and static conditions would favor stable contacts and close proximity between bacteria. Indeed, we observed that one *P*. *aeruginosa* PAO1 CDI system we studied is not able to mediate competition upon a defect in cell adhesion (Fig 3 and Fig 4).

It is now accepted that CDI systems are also involved in biofilm formation and self-recognition by inhibiting the incorporation of non-self competitors in the community. These mechanisms have been observed for *B*. *thailandensis* and *E*. *coli* EC93 CDI systems [8,10–12]. Our results demonstrate that *Pseudomonas* CdiA proteins also play a role in biofilm formation. Confirming the implication of the CDI systems in *Pseudomonas* biofilm architecture is a main goal of future studies. Interestingly, the CdiA proteins characterized in this study are among the five most abundant proteins found in the extracellular matrix of colony biofilms as well as in outer membrane vesicles (OMVs) [21]. OMVs are spherical structures derived from outer membranes of gram-negative bacteria [22], which have specific virulence-associated activities but are also implicated in cell-cell communication, horizontal gene transfer and competition with other organisms to promote bacterial colonization [23], activities surprisingly close to those assigned to CDI systems. CdiA-associated vesicles could be advantageous acting as bridging factors in biofilms and/or targeting distant bacteria in a competition context. While the role of OMVs in inter-bacterial interactions is well documented, no implication of CDI systems in this context has been described so far.

Most *Pseudomonas* genomes encode multiple CDI systems (Table 1) and in the case of *P*. *aeruginosa* species, our study indicates that CDI systems undergo the same regulatory pathway suggesting that each strain coordinately activates their CDI systems. The determination of the actor(s) implicated in cell inhibition is complex. Indeed, we found that *cdi* loci contain additional orphan *cdiA-CT/cdiI* modules located downstream to *cdiBAI* genes (S2 Table). Interestingly, the presence of such orphan modules is limited to the *P*. *aeruginosa* species. We showed that some of these modules are functional and have growth inhibition activities in *E*. *coli* but we have no clues on their synthesis under physiological conditions. Identified orphan *cdiA-CT* sequences lack initiation codon and RBS sequence indicating that they might not be produced in contrast to *cdiI* genes that have translation initiation signal. It is then conceivable that these *cdiI* genes are expressed as the orphan *cdiI*_*o1*_^*EC93*^ gene in *E*. *coli* EC93 [24]. The function of orphan toxin-immunity modules is unknown but they may represent a reservoir of diverse toxins or may be implicated in discerning kin versus non-kin within a population [25,26]. The co-existence of different *P*. *aeruginosa* lineages observed in chronic CF lung infections is in line with this concept [27]. A further complication in studying the polymicrobial interactions is that other factors contribute to inter-species competition such as type VI secretion systems (T6SS), which target microorganisms from the same or a different genus [28]. Interestingly, one of the *P*. *aeruginosa* T6SS, H1-T6SS, is also under the control of RsmA [29–31]. The co-activation of the T6SS and CDI system as anti-bacterial weapons in *P*. *aeruginosa* species would ensure a fitness advantage against other pathogens especially in lung infections of CF patients, where the bacteria are thought to reside within biofilm-like structures. In conclusion, the finding that a considerable number of strains encode diverse putative CDI systems suggests that these systems play a significant role in the social life of Pseudomonadaceae.

### Experimental procedures

#### Growth condition and strain constructions

Plasmids (listed in S1 Table) were maintained in *E*. *coli* strains and transferred into *P*. *aeruginosa* PAO1 strain by electroporation [32] or by tri-parental mating using the helper plasmid pRK2013 [33]. *P*. *aeruginosa* was cultured in solid or liquid Luria-Bertani (LB) medium supplemented with appropriate antibiotics: 30 μg/ml gentamycin (Gm), 50 μg.ml^-1^ carbenicillin (300 μg.ml^-1^ on plates), 2 mg.ml^-1^ streptomycin. M63 minimal medium (15 mM (NH_4_)_2_SO_4_, 22 mM KH_2_PO_4_, 40 mM K_2_HPO_4_, pH6.8) supplemented with 1 mM MgCl_2_, 0.5% casamino acids and 0.2% glucose was used for growth in static chambers.

The *P*. *aeruginosa* PAO1 *cdi* or *rsmA* genes deletion strains were engineered by allelic exchange. Briefly, the DNA regions upstream and downstream of the gene to be deleted were PCR amplified (Expand High Fidelity DNA polymerase, Roche), combined by overlapping extension PCR and cloned into the pKNG101 suicide vector [33]. The resulting plasmids were used to create deletion strains.

All Gm-resistant strains and green fluorescent protein (GFP)-tagged bacteria were created using respectively the pUC18T-mini-Tn7T-Gm and pUC18T-mini-Tn7T-Gm-*gfpmut3* [32]. The pJN107 plasmid was constructed by cloning the *Cla*I-*AraC*-P_*BAD*_-*EcoR*I fragment from pJN105 vector [34] into pBBR-MCS4 plasmid digested by *Cla*I/*EcoR*I [35]. *cdiI*_*PA0041*_ and *cdiI*_*PA2462*_ genes were amplified with an artificial Shine-Dalgarno (AAGAAG) and cloned into pJN107 using the SLIC method [36].

#### Isolation of total RNA and quantitative Real Time-PCR

qRT-PCR have been performed as previously described [37]. Briefly, total cellular RNAs from 1×10^10^ bacteria were isolated using the PureYield RNA Midiprep System (Promega) and contaminating DNA eliminated by TURBO DNase (Ambion) treatment. cDNA synthesis was performed on 1.6 μg of RNA using the SuperScriptIII first strand synthesis system (Invitrogen). The mRNAs level of *cdiA*_*PA2462*_ and *cdiA*_*PA0041*_ were determined using primers listed in S3 Table. To determine the amplification kinetics of each product during the quantitative reverse transcription, the fluorescence derived from the incorporation of EvaGreen into the double-stranded PCR products was measured at the end of each cycle using the SoFast EvaGreen Supermix (Biorad). The *16S* gene was used as control to normalize the results.

#### Adhesion and biofilm assays

GFP-tagged strains were cultured overnight in LB medium, then washed in M63 medium and inoculated to an OD_600_ of ~ 0.1 in 400 μl of M63 medium in chambered cover glass dishes (Thermo scientific) and incubated at 30°C for 8 h, washed 2 times with 1X PBS, overlaid with 400 μl of 1X PBS and imaged by CLSM with a olympus FV-1000 microscope. Biofilm assays were performed following the O’Toole protocol [38]. Briefly, overnight cultures were inoculated into LB at a final OD_600_ of 0.05. 200 μl of culture were added to 96-well polystyrene plates and incubated at 37°C over a 24h period. At the indicated times, cell in suspension were removed and 200 μl of 0.1% crystal violet was added to the wells and incubated for 10 min. Wells were washed three times with 1X PBS, then crystal violet was solubilized with 200 μl 95% ethanol and the absorbance was measured at 550 nm.

#### Growth competitions assays

Overnight cultures were washed, diluted to an OD_600_ of ~ 0.1 in LB medium and grown in static condition at 30°C to an OD_600_ ~ 0.3. Strains were mixed at an attacker to target cell ratio of 4 to 1 and 10 μl of mixed strains were used for determination of target initial CFU by plating on Gm-containing LB agar plates (t_0h_). 25 μl of the mix was spotted on 1.5% agar plate and incubated at 30°C for 24 h. Spotted bacteria were then resuspended in 500 μl LB and plated on Gm-containing LB agar plates to determine the target’s CFU after incubation with attacker (t_24h_). The viable target cells were calculated as the CFU.ml^-1^ (t_24h_)/CFU.ml^-1^ (t_0h_) ratio. Data were examined for significance using a two-tailed Student’s t-test. * 0.05<*p*-value<0.025; ** 0.025<*p*-value<0.01; *** *p*-value<0.01; ns: non-significant.

#### Vectors construction and toxicity assays in *E*. *coli* DH5α

CT domains delimited from their transition motif (S5 Fig) were cloned with the artificial AGGAGG Shine-Dalgarno sequence into the pBAD33 plasmid using *Sac*I and *Sal*I. The *cdiI* genes were PCR-amplified with a reverse primer encoding a 6xHis C-terminal tag and cloned into the pTrc99a plasmid using *Nco*I and *BamH*I. To assess CT-domain toxicity, bacteria were grown overnight with 0.5% glucose to repress CT-domain expression, washed twice in LB medium and diluted to an OD_600_ ~ 0.05 in LB with 1 mM IPTG to induce immunity protein production and 0.5% glucose or 1% arabinose to repress or induce CT-domain expression respectively. After 6 h of culture at 37°C, cells were washed twice in LB medium and plated onto LB agar plates supplemented with 0.5% glucose to determine the CFU/ml. Data were analyzed for significance using a two-tailed Student’s t-test. * 0.05<*p*-value<0.025; ** 0.025<*p*-value<0.01; *** *p*-value<0.01. Production of immunity proteins was verified in parallel by western-blot analyses by resuspending the bacterial pellet in 1X SDS-PAGE loading buffer. 0.1 OD_600_ units of boiled samples were separated on 18% SDS-PAGE gel, transferred onto nitrocellulose membranes and probed with primary mouse anti-pentaHis (Qiagen) or anti-EF-Tu (Hycult Biotech) antibodies and anti-mouse IgG conjugated to HRP (Sigma).

#### Vector construction and protein production in *E*. *coli* BL21(DE3)

The *cdiI*_*PA0041*_ gene was PCR amplified with a reverse primer encoding a 6xHis C-terminal tag and cloned into the pRSF-Duet1 vector using *Nco*I and *BamH*I. The CT domain of *cdiA*_*PA0041*_ was cloned into pRSF-Duet-CdiI_PA0041_ with *Nde*I and *Xho*I. *cdiI*_*PA2462*_, PCR-amplified with a reverse primer encoding a 6xHis C-terminal tag, was cloned into the pET19b vector with *Nco*I and *BamH*I.

Proteins were produced in *E*. *coli* BL21(DE3). Overnight cultures were diluted in LB medium to an OD_600_ ~ 0.05, grown at 37°C to OD_600_ ~ 0.5 and proteins produced for 2 h with 1 mM IPTG. The bacterial pellets were resuspended in lysis buffer (50 mM Tris-HCl pH8, 150 mM NaCl, 0.1% Triton X-100, 1X protease inhibitor cocktail (Complete EDTA-free; Roche), and 10% glycerol), lysed by sonication and centrifuged for 1 hr at 100,000×*g* at 4°C. Supernatants were loaded on Ni-NTA resin columns equilibrated in buffer A (50 mM Tris-HCl pH8, 300 mM NaCl, 0.1% Triton X-100 and 10% glycerol). After washing the resin with buffer A supplemented with 20 mM imidazole, CdiI_PA0041_-His_6_ and CdiI_PA2462_-His_6_ were eluted using a linear gradient of 50–400 mM imidazole in buffer A. Peak fractions were pooled, dialyzed against storage buffer (50 mM Tris-HCl pH7.5, 150 mM NaCl, 1 mM β-mercaptoethanol and 30% glycerol) and stored at -80°C. CdiA-CT_PA0041_ was eluted with denaturating buffer (buffer A supplemented with 6 M guanidine-HCl) and peak fractions were pooled, dialyzed twice against storage buffer and frozen to -80°C.

#### Pull down experiments

100 pmol of each protein were incubated at room temperature for 30 min. An aliquot of the mixture was removed to analyze the input fraction. Mixtures were then incubated 1 h at 4°C with Ni^2+^-NTA resin pre-equilibrated with buffer A. Unbound fraction was removed before washing the resin with buffer A supplemented with 25 mM imidazole. Bound complexes were eluted with buffer A supplemented with 300 mM imidazole. Prior 18% SDS-PAGE running, proteins were precipitated with 12% (w/v) trichloroacetic acid (TCA) and resuspended in 1X SDS-PAGE loading buffer.

## Supporting Information

S1 Fig Sequence identity of CdiA-CT_PA2462_ with MafB from *N*. *meningitidis* NEM8013. The 117 last C-terminal residues of PA2462 were aligned with the EndoU nuclease domain of MafB_MGI-1NEM8013_ (C9X2Z7) using ClustalW (1.8) multiple sequence alignment with default parameters and identical and similar residues were shaded with black or grey respectively using the BoxShade server.
(TIF)

S2 Fig Pairwise comparison of the genetic organization of the *PA2463-PA2463* and *PA0040-PA0041* loci. White triangles represent the predicted immunity genes non-annotated on the *P*. *aeruginosa* PAO1 genome. Sequence alignment was generated using Webact (http://www.webact.org/WebACT/home) and implemented in Easyfig 2.1 with no cutoff value. Blue vertical and orange blocks represent normal and inverted blast matches respectively, with a gradient scale showing the level of nucleotide identity. GC content greater and lower than 50% are represented in red and blue respectively. The orientation of region including *PA2463-PA2462* locus has been reversed for clarity.
(TIFF)

S3 Fig Analysis of *cdiA* gene expression under the minimal media growth conditions used to assay adhesion. The level of mRNA was measured by qRT-PCR in wild-type strain grown 7 h at 30°C in M63 medium under planktonic or static condition. For each gene, expression was normalized to *16S* expression and is shown relative to the planktonic condition level. Error bars represent the standard error of the mean from three independent experiments. Data were analyzed for significance using a two-tailed Student’s t-test. *** *p*-value<0.01.
(TIFF)

S4 Fig Production of immunity proteins in *E*. *coli* toxicity assay. After 6 h of culture with glucose (Glc) or arabinose (Ara), *E*. *coli* cell extracts containing indicated set of plasmids (A) and (B) were subjected to 18% SDS-PAGE and the production of CdiI-His_6_ and the cytoplasmic EF-Tu control were confirmed by western blot experiments using the anti-pentaHis (top panels) and anti-EF-Tu monoclonal antibodies (bottom panels). Molecular weight markers (in kDa) are indicated on the right.
(TIFF)

S5 Fig Representation of classes based on amino acid sequence alignments. Protein sequences were aligned using ClustalW (1.8) multiple sequence alignment, with default parameters and shaded using the BoxShade server. Identical and similar residues are shaded with black or grey respectively. Numbers represent the amino acid position of each CdiA proteins.
(TIFF)

S1 Table Plasmids used in this study. (TIFF)

S2 Table List of orphan modules. (TIFF)

S3 Table List of primers used in qRT-PCR. (TIFF)

## References

[1] Jamet A, Nassif X. New players in the toxin field: polymorphic toxin systems in bacteria. mBio. 2015;6: e00285–00215. 10.1128/mBio.00285-15 25944858 PMC4436062

[2] Zhang D, de Souza RF, Anantharaman V, Iyer LM, Aravind L. Polymorphic toxin systems: Comprehensive characterization of trafficking modes, processing, mechanisms of action, immunity and ecology using comparative genomics. Biol Direct. 2012;7: 18 10.1186/1745-6150-7-18 22731697 PMC3482391

[3] Aoki SK. Contact-Dependent Inhibition of Growth in *Escherichia coli*. Science. 2005;309: 1245–1248. 10.1126/science.1115109 16109881

[4] Mazar J, Cotter PA. New insight into the molecular mechanisms of two-partner secretion. Trends Microbiol. 2007;15: 508–515. 10.1016/j.tim.2007.10.005 17988872

[5] Aoki SK, Diner EJ, de Roodenbeke C t’Kint, Burgess BR, Poole SJ, Braaten BA, et al A widespread family of polymorphic contact-dependent toxin delivery systems in bacteria. Nature. 2010;468: 439–442. 10.1038/nature09490 21085179 PMC3058911

[6] Morse RP, Nikolakakis KC, Willett JLE, Gerrick E, Low DA, Hayes CS, et al Structural basis of toxicity and immunity in contact-dependent growth inhibition (CDI) systems. Proc Natl Acad Sci. 2012;109: 21480–21485. 10.1073/pnas.1216238110 23236156 PMC3535622

[7] Beck CM, Morse RP, Cunningham DA, Iniguez A, Low DA, Goulding CW, et al CdiA from *Enterobacter cloacae* Delivers a Toxic Ribosomal RNase into Target Bacteria. Structure. 2014;22: 707–718. 10.1016/j.str.2014.02.012 24657090 PMC4016183

[8] Anderson MS, Garcia EC, Cotter PA. The *Burkholderia bcpAIOB* Genes Define Unique Classes of Two-Partner Secretion and Contact Dependent Growth Inhibition Systems. Guttman DS, editor. PLoS Genet. 2012;8: e1002877 10.1371/journal.pgen.1002877 22912595 PMC3415462

[9] Nikolakakis K, Amber S, Wilbur JS, Diner EJ, Aoki SK, Poole SJ, et al The toxin/immunity network of *Burkholderia pseudomallei* contact-dependent growth inhibition (CDI) systems. Mol Microbiol. 2012;84: 516–529. 10.1111/j.1365-2958.2012.08039.x 22435733 PMC3331888

[10] Garcia EC, Anderson MS, Hagar JA, Cotter PA. BcpA mediates biofilm formation independently of interbacterial contact-dependent growth inhibition: CDI system protein-mediated biofilm. Mol Microbiol. 2013;89: 1213–1225. 10.1111/mmi.12339 23879629 PMC3786370

[11] Ruhe ZC, Townsley L, Wallace AB, King A, Van der Woude MW, Low DA, et al CdiA promotes receptor-independent intercellular adhesion. Mol Microbiol. 2015; 10.1111/mmi.13114 PMC4694591 26135212

[12] Anderson MS, Garcia EC, Cotter PA. Kind Discrimination and Competitive Exclusion Mediated by Contact-Dependent Growth Inhibition Systems Shape Biofilm Community Structure. Mougous JD, editor. PLoS Pathog. 2014;10: e1004076 10.1371/journal.ppat.1004076 24743836 PMC3990724

[13] Ghequire MGK, De Mot R. Ribosomally encoded antibacterial proteins and peptides from *Pseudomonas*. FEMS Microbiol Rev. 2014;38: 523–568. 10.1111/1574-6976.12079 24923764

[14] Jamet A, Jousset AB, Euphrasie D, Mukorako P, Boucharlat A, Ducousso A, et al A New Family of Secreted Toxins in Pathogenic *Neisseria* Species. Skaar EP, editor. PLoS Pathog. 2015;11: e1004592 10.1371/journal.ppat.1004592 25569427 PMC4287609

[15] Brencic A, Lory S. Determination of the regulon and identification of novel mRNA targets of *Pseudomonas aeruginosa* RsmA. Mol Microbiol. 2009;72: 612–632. 10.1111/j.1365-2958.2009.06670.x 19426209 PMC5567987

[16] Vakulskas CA, Potts AH, Babitzke P, Ahmer BMM, Romeo T. Regulation of Bacterial Virulence by Csr (Rsm) Systems. Microbiol Mol Biol Rev MMBR. 2015;79: 193–224. 10.1128/MMBR.00052-14 25833324 PMC4394879

[17] Amitai G, Belenkiy O, Dassa B, Shainskaya A, Pietrokovski S. Distribution and function of new bacterial intein-like protein domains. Mol Microbiol. 2003;47: 61–73. 12492854 10.1046/j.1365-2958.2003.03283.x

[18] Zhang D, Iyer LM, Aravind L. A novel immunity system for bacterial nucleic acid degrading toxins and its recruitment in various eukaryotic and DNA viral systems. Nucleic Acids Res. 2011;39: 4532–4552. 10.1093/nar/gkr036 21306995 PMC3113570

[19] Hartley RW. Barnase and barstar: two small proteins to fold and fit together. Trends Biochem Sci. 1989;14: 450–454. 2696173 10.1016/0968-0004(89)90104-7

[20] Perler FB. Protein splicing of inteins and hedgehog autoproteolysis: structure, function, and evolution. Cell. 1998;92: 1–4. 10.1016/s0092-8674(00)80892-2 9489693

[21] Toyofuku M, Roschitzki B, Riedel K, Eberl L. Identification of proteins associated with the *Pseudomonas aeruginosa* biofilm extracellular matrix. J Proteome Res. 2012;11: 4906–4915. 10.1021/pr300395j 22909304

[22] Kulp A, Kuehn MJ. Biological functions and biogenesis of secreted bacterial outer membrane vesicles. Annu Rev Microbiol. 2010;64: 163–184. 10.1146/annurev.micro.091208.073413 20825345 PMC3525469

[23] Ellis TN, Kuehn MJ. Virulence and immunomodulatory roles of bacterial outer membrane vesicles. Microbiol Mol Biol Rev MMBR. 2010;74: 81–94. 10.1128/MMBR.00031-09 20197500 PMC2832350

[24] Poole SJ, Diner EJ, Aoki SK, Braaten BA, t’ Kint de Roodenbeke C, Low DA, et al Identification of Functional Toxin/Immunity Genes Linked to Contact-Dependent Growth Inhibition (CDI) and Rearrangement Hotspot (Rhs) Systems. Achtman M, editor. PLoS Genet. 2011;7: e1002217 10.1371/journal.pgen.1002217 21829394 PMC3150448

[25] Koskiniemi S, Lamoureux JG, Nikolakakis KC, t’ Kint de Roodenbeke C, Kaplan MD, Low DA, et al Rhs proteins from diverse bacteria mediate intercellular competition. Proc Natl Acad Sci. 2013;110: 7032–7037. 10.1073/pnas.1300627110 23572593 PMC3637788

[26] Koskiniemi S, Garza-Sánchez F, Sandegren L, Webb JS, Braaten BA, Poole SJ, et al Selection of Orphan Rhs Toxin Expression in Evolved *Salmonella enterica* Serovar Typhimurium. Søgaard-Andersen L, editor. PLoS Genet. 2014;10: e1004255 10.1371/journal.pgen.1004255 24675981 PMC3967940

[27] Williams D, Evans B, Haldenby S, Walshaw MJ, Brockhurst MA, Winstanley C, et al Divergent, Coexisting, *Pseudomonas aeruginosa* Lineages in Chronic Cystic Fibrosis Lung Infections. Am J Respir Crit Care Med. 2015; 10.1164/rccm.201409-1646OC PMC4407486 25590983

[28] Hood RD, Singh P, Hsu F, Güvener T, Carl MA, Trinidad RRS, et al A type VI secretion system of *Pseudomonas aeruginosa* targets a toxin to bacteria. Cell Host Microbe. 2010;7: 25–37. 10.1016/j.chom.2009.12.007 20114026 PMC2831478

[29] Mougous JD, Cuff ME, Raunser S, Shen A, Zhou M, Gifford CA, et al A virulence locus of *Pseudomonas aeruginosa* encodes a protein secretion apparatus. Science. 2006;312: 1526–1530. 10.1126/science.1128393 16763151 PMC2800167

[30] Wei X, Huang X, Tang L, Wu D, Xu Y. Global control of GacA in secondary metabolism, primary metabolism, secretion systems, and motility in the rhizobacterium *Pseudomonas aeruginosa* M18. J Bacteriol. 2013;195: 3387–3400. 10.1128/JB.00214-13 23708134 PMC3719553

[31] Li K, Xu C, Jin Y, Sun Z, Liu C, Shi J, et al SuhB is a regulator of multiple virulence genes and essential for pathogenesis of *Pseudomonas aeruginosa*. mBio. 2013;4: e00419–00413. 10.1128/mBio.00419-13 24169572 PMC3809559

[32] Choi K-H, Schweizer HP. mini-Tn7 insertion in bacteria with single *attTn7* sites: example *Pseudomonas aeruginosa*. Nat Protoc. 2006;1: 153–161. 10.1038/nprot.2006.24 17406227

[33] Kaniga K, Delor I, Cornelis GR. A wide-host-range suicide vector for improving reverse genetics in gram-negative bacteria: inactivation of the *blaA* gene of *Yersinia enterocolitica*. Gene. 1991;109: 137–141. 1756974 10.1016/0378-1119(91)90599-7

[34] Newman JR, Fuqua C. Broad-host-range expression vectors that carry the L-arabinose-inducible *Escherichia coli araBAD* promoter and the araC regulator. Gene. 1999;227: 197–203. 10023058 10.1016/s0378-1119(98)00601-5

[35] Kovach ME, Elzer PH, Hill DS, Robertson GT, Farris MA, Roop RM, et al Four new derivatives of the broad-host-range cloning vector pBBR1MCS, carrying different antibiotic-resistance cassettes. Gene. 1995;166: 175–176. 8529885 10.1016/0378-1119(95)00584-1

[36] Jeong J-Y, Yim H-S, Ryu J-Y, Lee HS, Lee J-H, Seen D-S, et al One-step sequence- and ligation-independent cloning as a rapid and versatile cloning method for functional genomics studies. Appl Environ Microbiol. 2012;78: 5440–5443. 10.1128/AEM.00844-12 22610439 PMC3416421

[37] Faure LM, Llamas MA, Bastiaansen KC, de Bentzmann S, Bigot S. Phosphate starvation relayed by PhoB activates the expression of the *Pseudomonas aeruginosa* σvreI ECF factor and its target genes. Microbiol Read Engl. 2013;159: 1315–1327. 10.1099/mic.0.067645-0 23657684

[38] O’Toole GA. Microtiter dish biofilm formation assay. J Vis Exp JoVE. 2011; 10.3791/2437 PMC3182663 21307833

