# Correction: Functional Characterization of *Pseudomonas* Contact Dependent Growth Inhibition (CDI) Systems

**DOI:** 10.1371/journal.pone.0150538

**Published:** 2016-02-25

**Authors:** Chryslène Mercy, Bérengère Ize, Suzana P. Salcedo, Sophie de Bentzmann, Sarah Bigot

There is an error in Table 1. Lines 29 to 49 are incorrectly duplicated. Please see the corrected Table 1 here.

**Table 1:** predicted *Pseudomonas* CDI-encoding locus

Strain	CdiB	CdiA / CT domain^a^	motif	CdiI*	Stop-Start /lenght^b^	Class
*P*. *syringae* pv.tomato DC3000	PSPTO_3230	PSPTO_3229	PT-HINT	CdiI- PSPTO_3229*	**TAG**CTATG /132	I
*P*. *fluorescens* A506	PflA506_0158	PflA506_0159	PT-HINT/DUF637	PflA506_0160		I
*P*. *fluorescens* SBW25	PFLU_3192	PFLU_3191 / Tox-REase-7 (PF15649)	PT-HINT	PFLU_3190		I
*P*. *protegens* Pf-5	PFL_1551	PFL_1552	PT-HINT/DUF637	CdiI-PFL_1552*	**TGA**TTTATG /133	I
*P*. *fluorescens* SBW25	PFLU_0148	PFLU_0148	PT-VENN	CdiI-PFLU_0148*	ATGAAAAAG**TGA** /72	II
*P*. *fluorescens* SBW25	PFLU_3246	PFLU_3246 / Tox-HNH-HHH (PF15637)	PT-VENN	CdiI-PFLU_3246*	**TA****A**TG /143	II
*P*. *aeruginosa* NCGM2.S1	NCGM2_0040	NCGM2_0041 / MafB19-deam (PF14437)	PT-VENN	CdiI-NCGM2_0041*	AATA**TG****A** /133	II
*P*. *fulva* 12-X	Psefu_0120	Psefu_0121/ Tox-REase-7 (PF15649)	none	Psefu_0122		III
*P*. *fluorescens* F113	PSF113_0792	PSF113_0793 / AHH (PF14412)	none	PSF113_0794		III
*P*. *fluorescens* A506	PflA506_2790	PflA506_2789	none	CdiI-PflA506_2789*	**TAG**AGAAATTTTATATG /68	III
*P*. *entomophila* L48	PSEEN_3947	PSEEN_3946	DUF637	CdiI-PSEEN_3946*	GCAG**TG****A**A /129	IV
*P*. *brassicacearum* DF41	CD58_03745	CD58_03750 / Ribonuclease/ribotoxin (IPR016191)	DUF637	CD58_03755		IV
*P*. *protegens* CHA0	PFLCHA0_c15900	PFLCHA0_c15910	DUF637	CdiI-PFLCHA0_c15910*	**TAG**AGGAGGACGGTTATG /160	IV
*P*. *aeruginosa* PAO1	PA2463	PA2462 /RNAse EndoU fold (PF14436)	none	CdiI- PA2462*	**TAG**GTCTTTTATG /84	V
*P*. *aeruginosa* PA14	PA14_32780	PA14_32790	DUF637	CdiI-PA14_32790*	AG**TG****A**A /113	V
*P*. *aeruginosa* PA7	PSPA7_2776	PSPA7_2777 / CDI_toxin_Bp1026b_like	DUF637	CdiI- PSPA7_2777*	GA**TG****A**GC /130	V
*P*. *aeruginosa* LESB58	PALES_2831	PALES_28341 / Toxin deaminase (PF14424)	none	CdiI-PALES_28341*	G**TA****A**TGG /117	V
*P*. *aeruginosa* PAO1	PA0040	PA0041 / CDI_toxin_Bp1026b_like	DUF637	CdiI-PA0041*	C**TGA**TTATG /84	V
*P*. *aeruginosa* PA7	PSPA7_0042	PSPA7_0044	DUF637	PSPA7_0045		V
*P*. *aeruginosa* LESB58	PALES_00391	PALES_00401	DUF637	CdiI- PALES_00401*	AAAA**TG****A**TT /106	V
*P*. *aeruginosa* PACS2	PaerPA_01000042	PaerPA_01000043 /Toxin_49 (PF15529)	DUF637	CdiI- PaerPA_01000043*	CA**TG****A**A /156	V
*P*. *aeruginosa* PA14	PA14_00490	PA14_00510	DUF637	CdiI-PA14_00510*	C**TG****A**TGA/155	V
*P*. *aeruginosa* NCGM2.S1	NCGM2_3465	NCGM2_3464	DUF637	CdiI- NCGM2_3464*	**TGA**GTTAATATG /106	V
*P*. *aeruginosa* DK2	PADK2_00205	PADK2_00210 / Pyocin large subunit (COG5529)	DUF637	CdiI-PADK2_00210*	**TAA**GGAGAGCCCAATG /80	V

^a^ CT domain: identified conserved domains using Interproscan5 program and NCBI database

^b^ stop codon of *cdiA* gene and start codon of *cdiI* gene are in bold and underlined respectively, number indicate the length of the predicted CdiI protein

*: non-annotated ORF encoding potential CdiI proteins
