# Supplementary figures and images for: Functional Characterization of Pseudomonas Contact Dependent Growth Inhibition (CDI) Systems
Source: PLoS One. 2016 Jan 25;11(1):e0147435. doi: 10.1371/journal.pone.0147435 (PMC4725963; doi:10.1371/journal.pone.0147435)

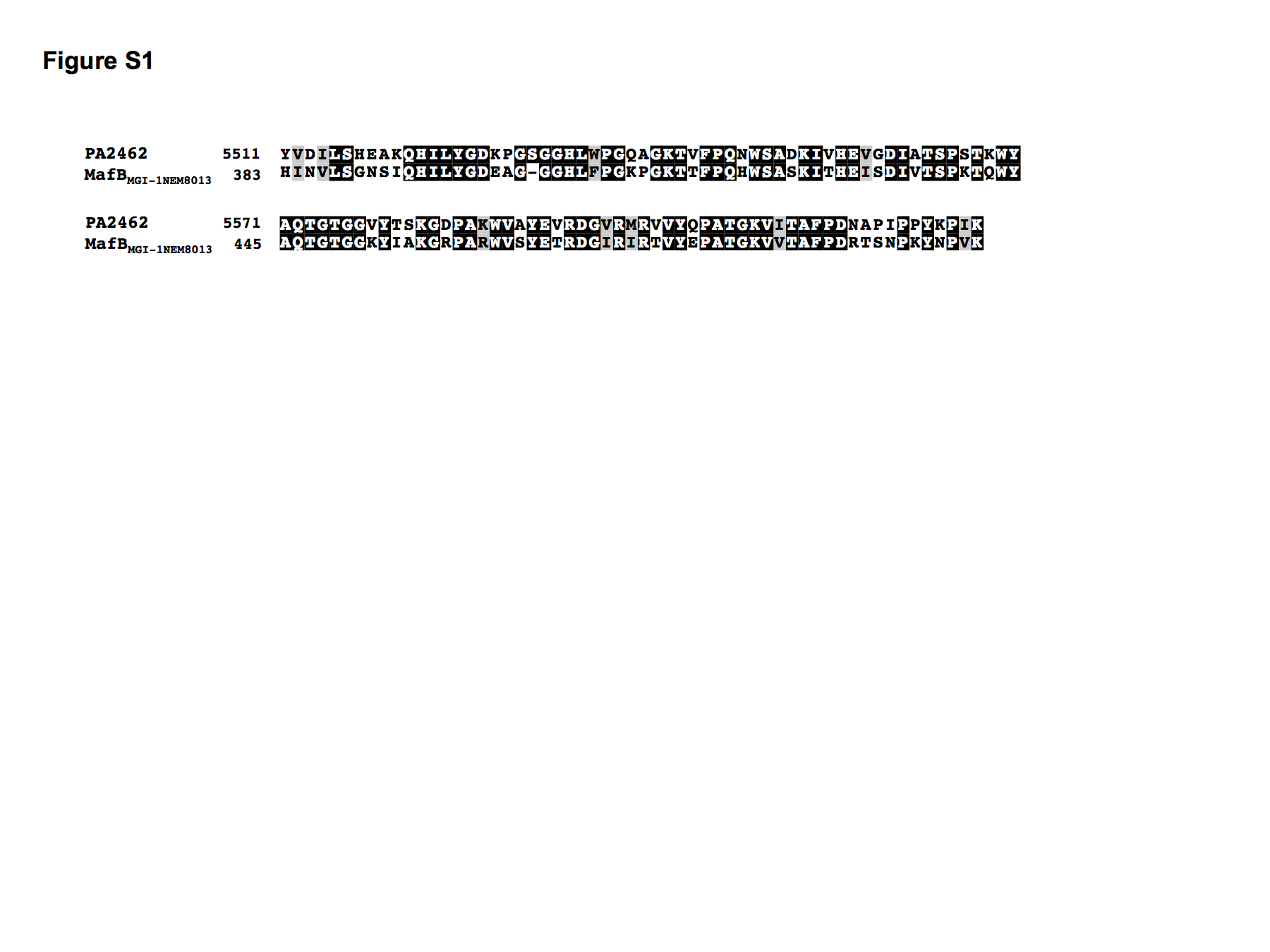

Supplement: S1 Fig — The 117 last C-terminal residues of PA2462 were aligned with the EndoU nuclease domain of MafBMGI-1NEM8013 (C9X2Z7) using ClustalW (1.8) multiple sequence alignment with default parameters and identical and similar residues were shaded with black or grey respectively using the BoxShade server. (TIF) [file pone.0147435.s001.tif]

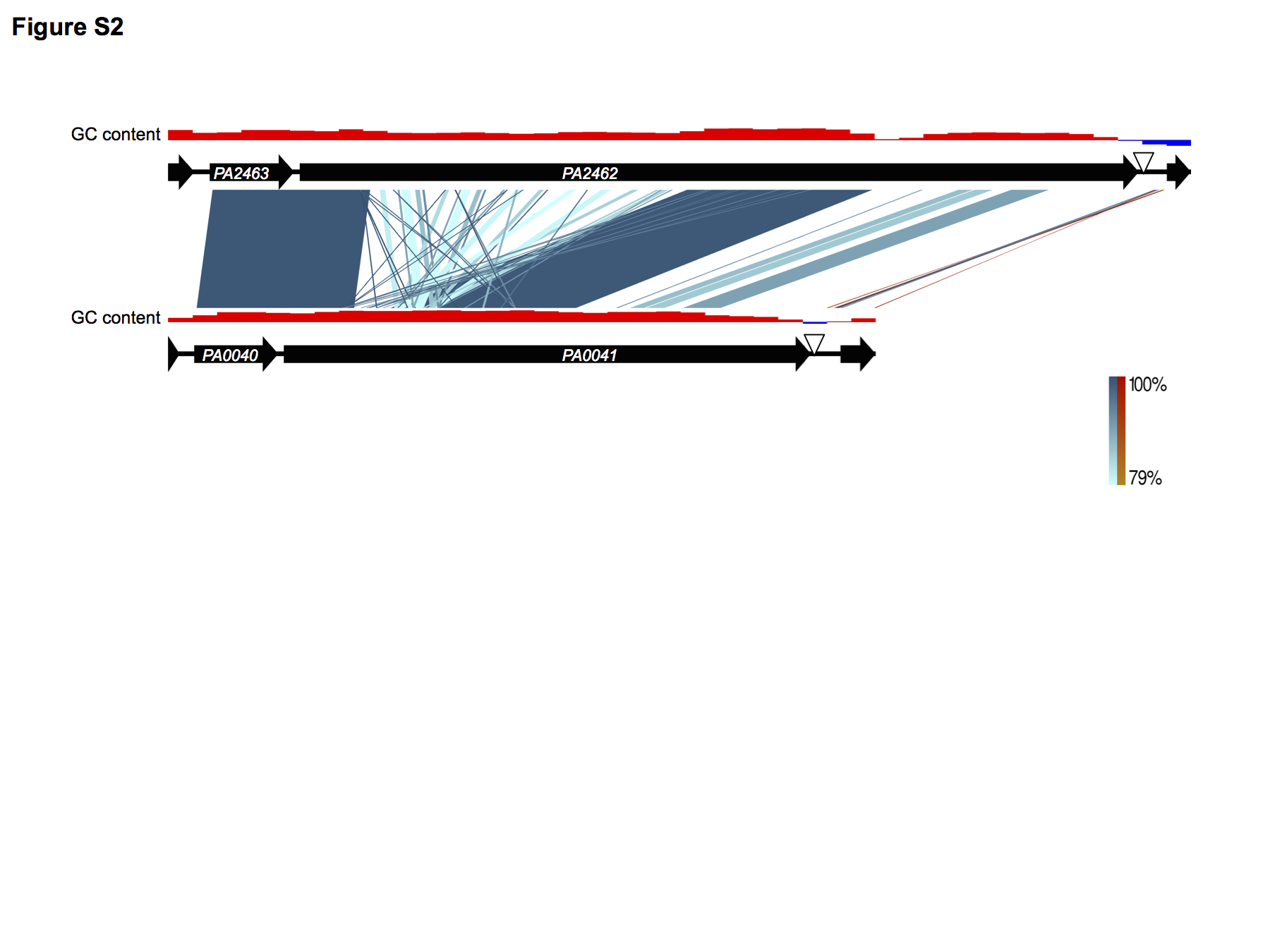

Supplement: S2 Fig — White triangles represent the predicted immunity genes non-annotated on the P. aeruginosa PAO1 genome. Sequence alignment was generated using Webact (http://www.webact.org/WebACT/home) and implemented in Easyfig 2.1 with no cutoff value. Blue vertical and orange blocks represent normal and inverted blast matches respectively, with a gradient scale showing the level of nucleotide identity. GC content greater and lower than 50% are represented in red and blue respectively. The orientation of region including PA2463-PA2462 locus has been reversed for clarity. (TIFF) [file pone.0147435.s002.tiff]

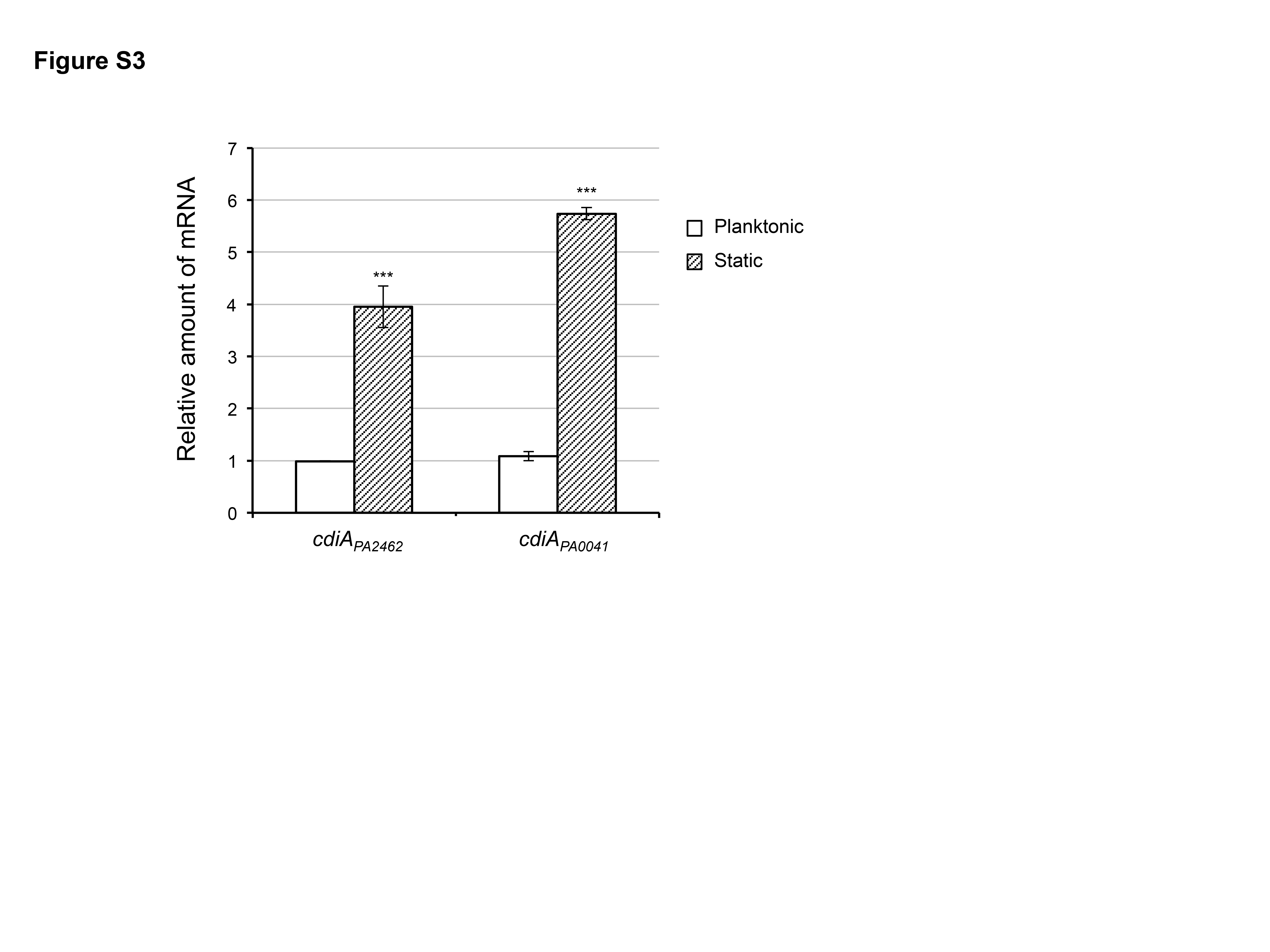

Supplement: S3 Fig — The level of mRNA was measured by qRT-PCR in wild-type strain grown 7 h at 30°C in M63 medium under planktonic or static condition. For each gene, expression was normalized to 16S expression and is shown relative to the planktonic condition level. Error bars represent the standard error of the mean from three independent experiments. Data were analyzed for significance using a two-tailed Student’s t-test. *** p-value<0.01. (TIFF) [file pone.0147435.s003.tiff]

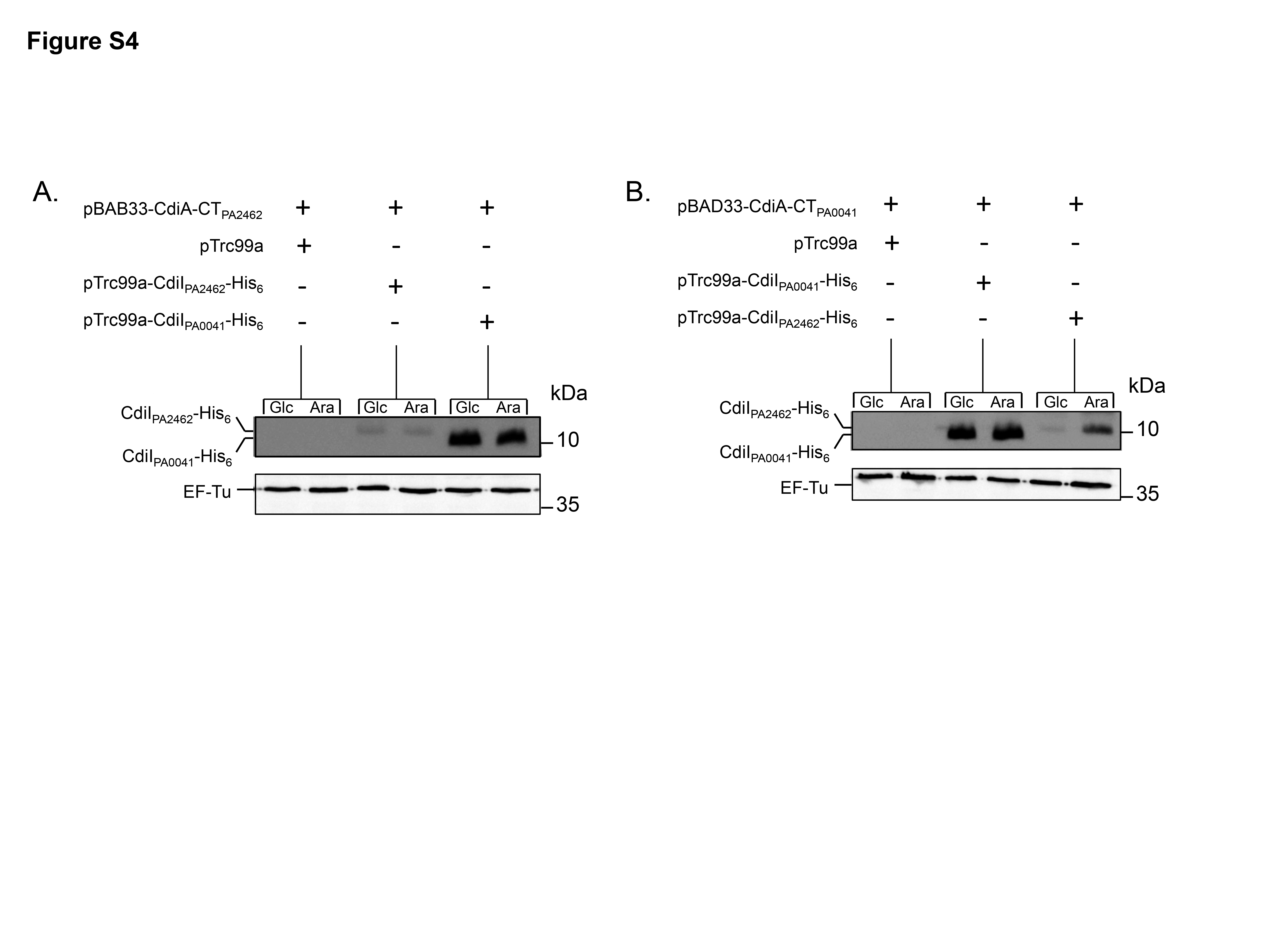

Supplement: S4 Fig — After 6 h of culture with glucose (Glc) or arabinose (Ara), E. coli cell extracts containing indicated set of plasmids (A) and (B) were subjected to 18% SDS-PAGE and the production of CdiI-His6 and the cytoplasmic EF-Tu control were confirmed by western blot experiments using the anti-pentaHis (top panels) and anti-EF-Tu monoclonal antibodies (bottom panels). Molecular weight markers (in kDa) are indicated on the right. (TIFF) [file pone.0147435.s004.tiff]

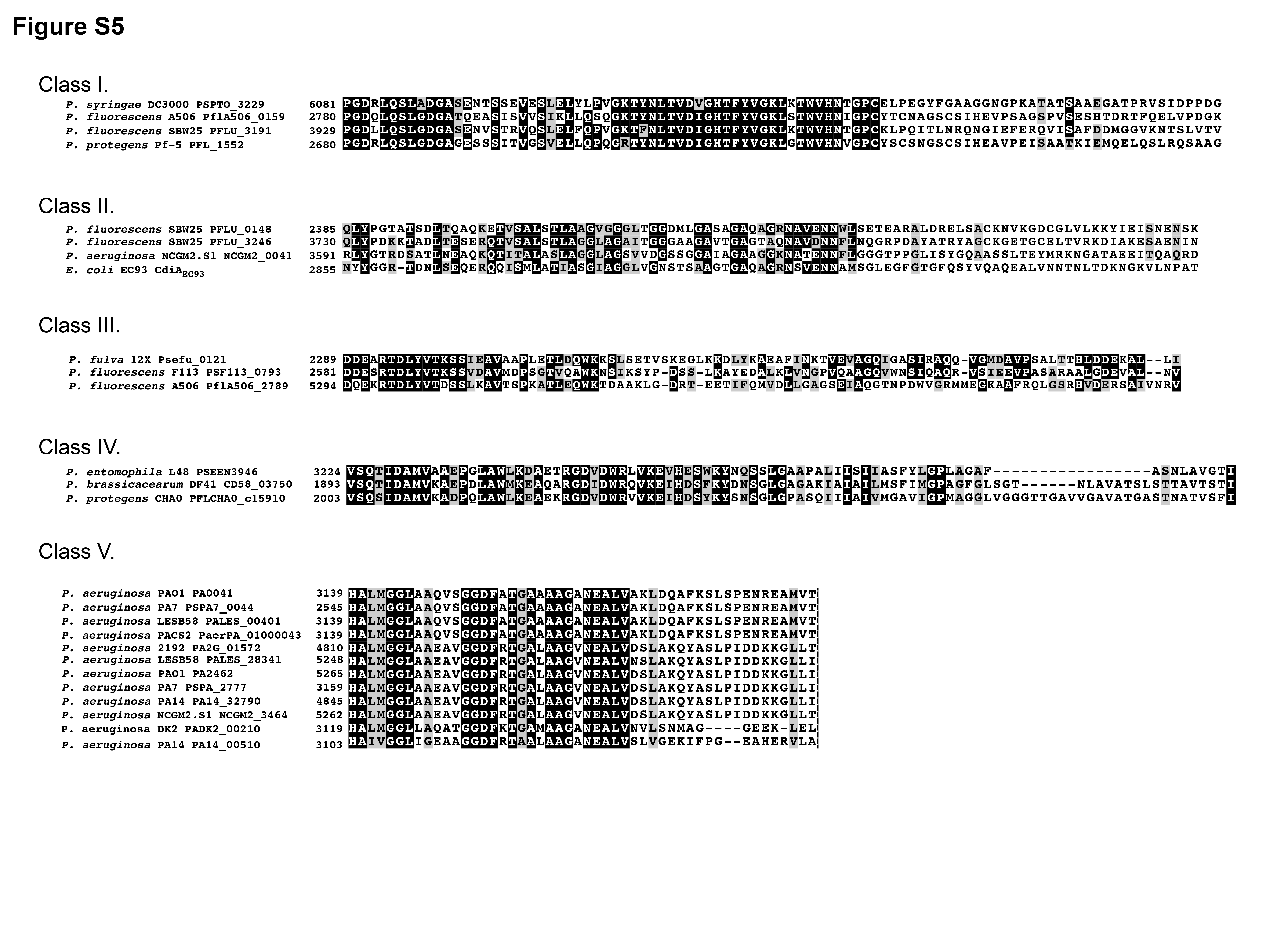

Supplement: S5 Fig — Protein sequences were aligned using ClustalW (1.8) multiple sequence alignment, with default parameters and shaded using the BoxShade server. Identical and similar residues are shaded with black or grey respectively. Numbers represent the amino acid position of each CdiA proteins. (TIFF) [file pone.0147435.s005.tiff]

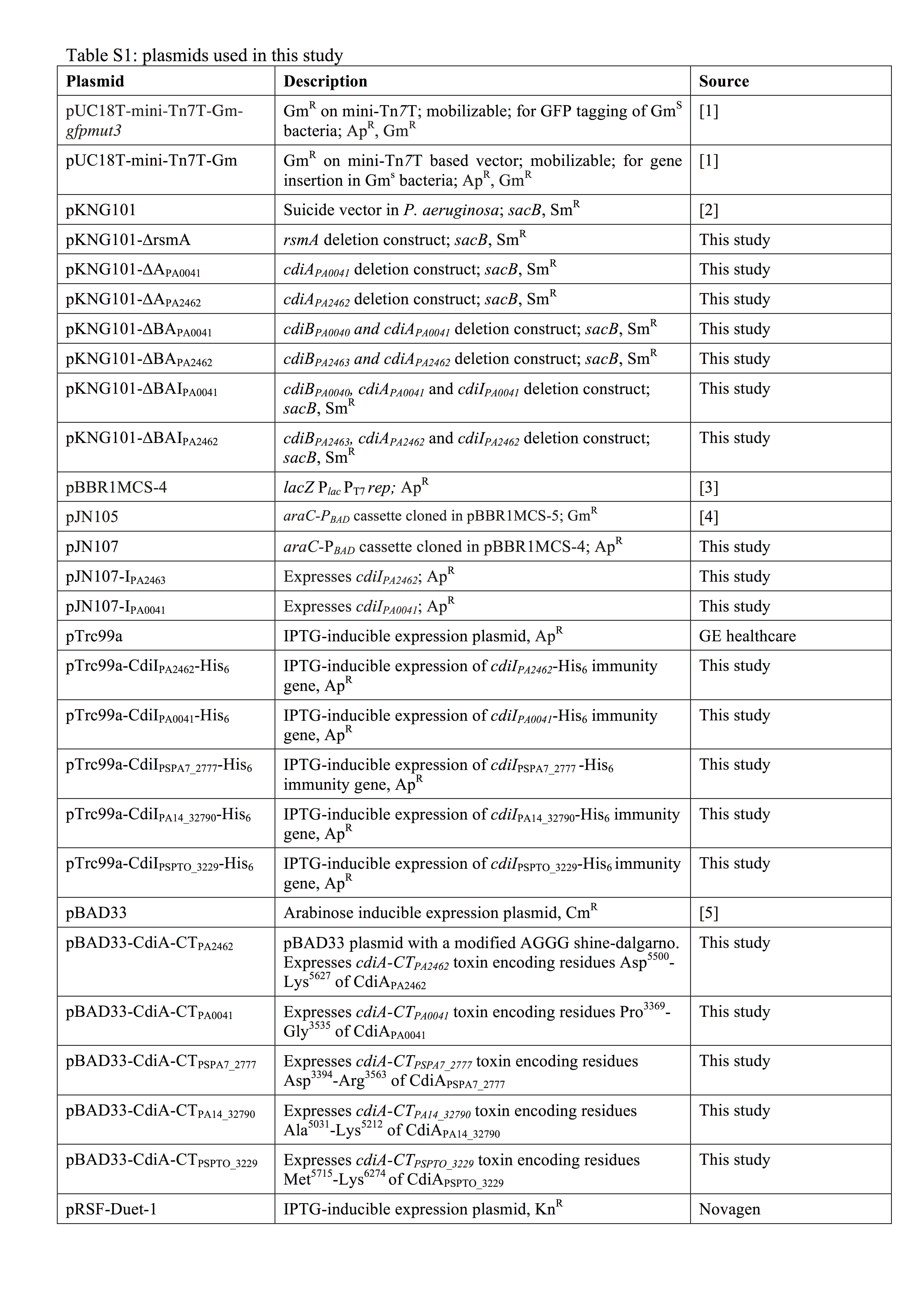

Supplement: S1 Table — (TIFF) [file pone.0147435.s006.tiff]

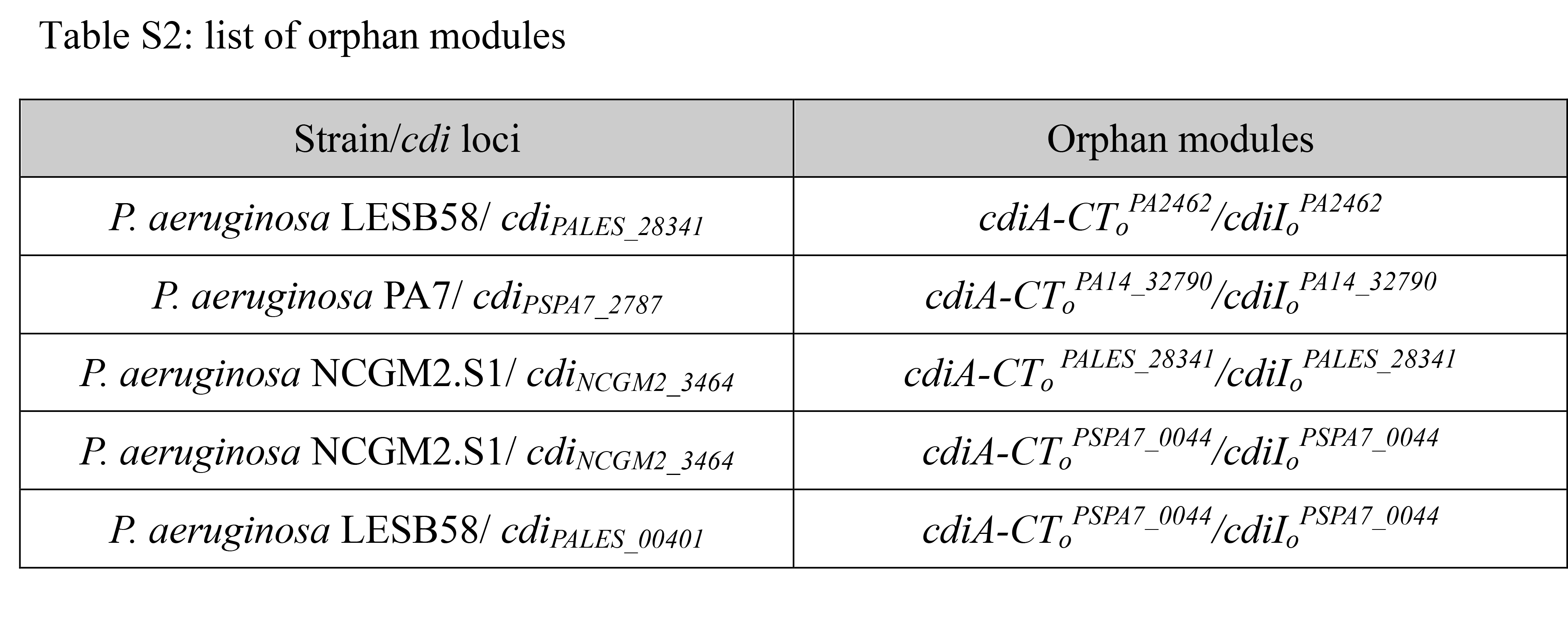

Supplement: S2 Table — (TIFF) [file pone.0147435.s007.tiff]

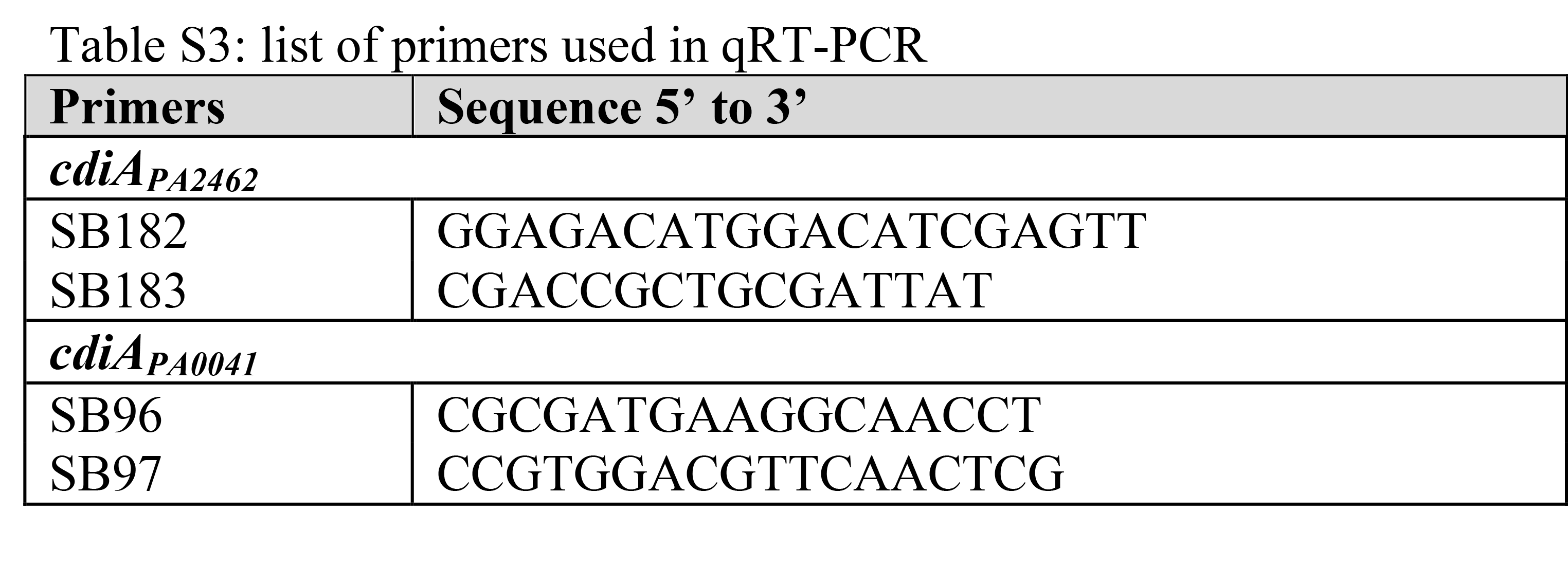

Supplement: S3 Table — (TIFF) [file pone.0147435.s008.tiff]
